# Divergent Proteome Reactivity Influences Arm-Selective Activation of the Unfolded Protein Response by Pharmacological Endoplasmic Reticulum Proteostasis Regulators

**DOI:** 10.1021/acschembio.3c00042

**Published:** 2023-07-31

**Authors:** Gabriel M. Kline, Ryan J. Paxman, Chung-Yon Lin, Nicole Madrazo, Leonard Yoon, Julia M. D. Grandjean, Kyunga Lee, Karina Nugroho, Evan T. Powers, R. Luke Wiseman, Jeffery W. Kelly

**Affiliations:** † Department of Chemistry, The Scripps Research Institute, La Jolla, California 92037, United States; ‡ Department of Molecular Medicine, The Scripps Research Institute, La Jolla, California 92037, United States; § The Skaggs Institute for Chemical Biology, The Scripps Research Institute, La Jolla, California 92037, United States

## Abstract

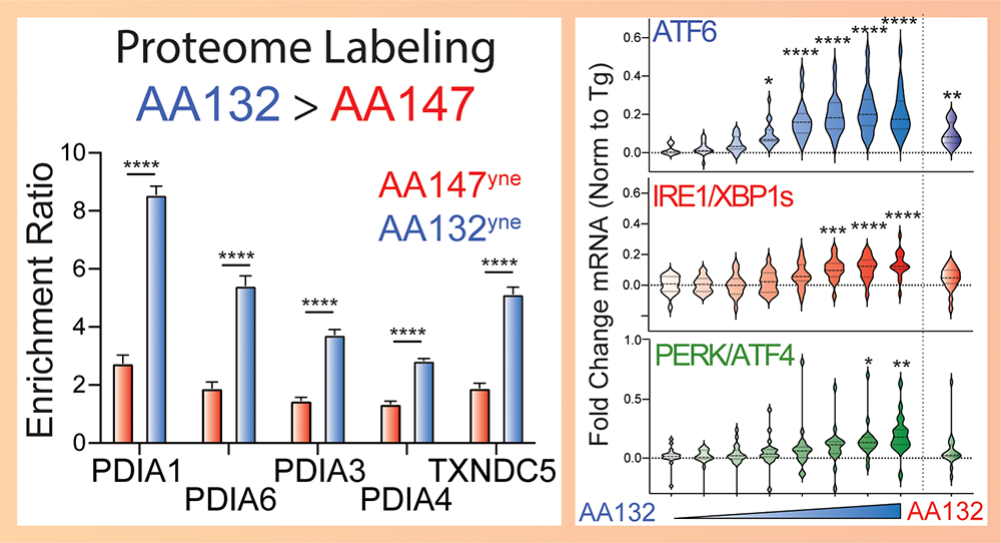

Pharmacological activation of the activating transcription factor 6 (ATF6) arm of the unfolded protein response (UPR) has proven useful for ameliorating proteostasis deficiencies in cellular and mouse models of numerous etiologically diverse diseases. Previous high-throughput screening efforts identified the small molecule AA147 as a potent and selective ATF6 activating compound that operates through a mechanism involving metabolic activation of its 2-amino-*p*-cresol substructure affording a quinone methide, which then covalently modifies a subset of endoplasmic reticulum (ER) protein disulfide isomerases (PDIs). Another compound identified in this screen, AA132, also contains a 2-amino-*p*-cresol moiety; however, this compound showed less transcriptional selectivity, instead globally activating all three arms of the UPR. Here, we show that AA132 activates global UPR signaling through a mechanism analogous to that of AA147, involving metabolic activation and covalent modification of proteins including multiple PDIs. Chemoproteomic-enabled analyses show that AA132 covalently modifies PDIs to a greater extent than AA147. However, the extent of PDI labeling by AA147 approaches a plateau more rapidly than PDI labeling by AA132. These observations together suggest that AA132 can access a larger pool of proteins for covalent modification, possibly because its activated form is less susceptible to quenching than activated AA147. In other words, the lower reactivity of activated AA132 allows it to persist longer and modify more PDIs in the cellular environment. Collectively, these results suggest that AA132 globally activates the UPR through increased engagement of ER PDIs. Consistent with this, reducing the cellular concentration of AA132 decreases PDI modifications and enables selective ATF6 activation. Our results highlight the relationship between metabolically activatable-electrophile stability, ER proteome reactivity, and the transcriptional response observed with the enaminone chemotype of ER proteostasis regulators, enabling continued development of next-generation ATF6 activating compounds.

## Introduction

The unfolded protein response (UPR) is an endoplasmic reticulum (ER) stress-responsive signaling pathway that corrects imbalances in ER protein homeostasis (proteostasis) caused by an ER stress.^1−3^ The UPR functions by simultaneously expanding ER folding capacity and restricting ER protein flux to restore ER proteostasis through both transcriptional and non-transcriptional responses.^1,3,4^ Activation of the UPR occurs downstream of three resident ER stress sensors: PKR-like endoplasmic reticulum kinase (PERK), inositol-requiring enzyme 1 (IRE1), and activating transcription factor 6 (ATF6). Transcription factors regulated downstream of ATF6 and IRE1, ATF6f and XBP1s, respectively, induce expression of numerous protective genes that remodel biological pathways involved in cellular metabolism, redox regulation, and ER proteostasis.^1,3,4^ This transcriptional remodeling functions to alleviate the ER stress by adapting cellular physiology to pathologic ER insults. While persistent activation of the IRE1 and PERK arms of the UPR have been associated with pathologic consequences, constitutive ATF6 activation to physiologically relevant levels has not generally been found to be detrimental in mammalian cell culture or mouse models.^5−10^ As such, genetic and pharmacological activation of ATF6 signaling has proven beneficial in mitigating pathological conditions resulting from proteostasis imbalances in numerous disease models.^11−16^ This suggests that ATF6 is an attractive therapeutic target to intervene in etiologically diverse diseases.^4,5,17,18^

**Figure 1:**
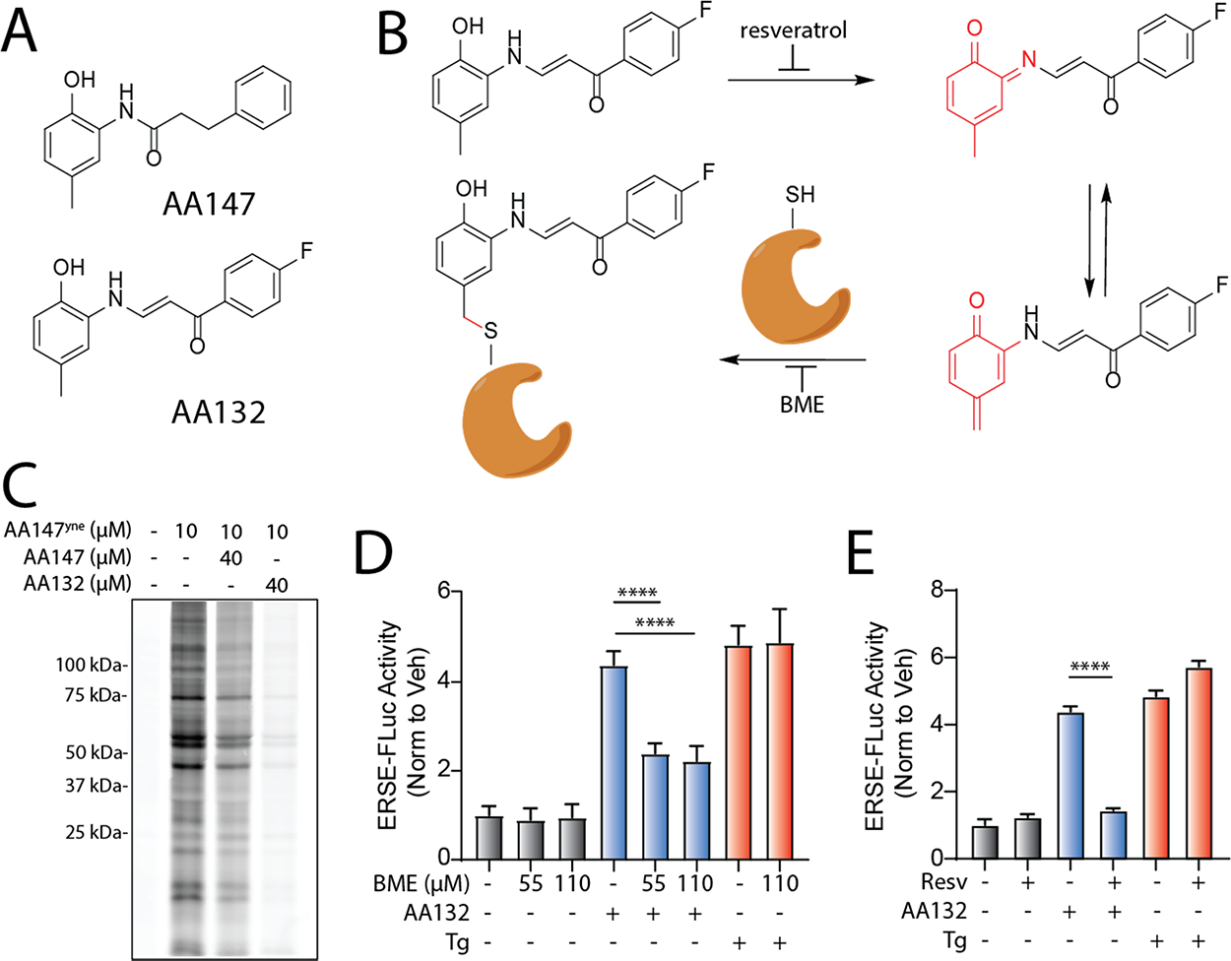
AA132 activates ATF6 signaling pathways through a mechanism involving metabolic activation and covalent protein modification. (A) Structures of AA132 and AA147. (B) Mechanism of AA132 metabolic activation and covalent protein modification. (C) Representative SDS-PAGE gel of Cy5-conjugated proteins from HEK293T cells treated for 4 h with vehicle (0.1% DMSO), AA147^yne^ (10 μM), the combination of AA147^yne^ (10 μM) and AA147 (40 μM), or the combination of AA147^yne^ (10 μM) and AA132 (40 μM). (D) Bar graph showing the activation of the ERSE.FLuc ATF6 reporter in HEK293T cells treated with AA132 (10 μM) or thapsigargin (Tg, 500 nM) ± β-mercaptoethanol (BME; 55 μM or 110 μM) for 18 h. Error bars show SEM for *N* = >3 replicates. ***p < 0.001, ****p < 0.0001. (E) Bar graph showing the activation of the ERSE.FLuc ATF6 reporter in HEK293T cells treated with AA132 (10 μM) or Tg (500 nM) ± resveratrol (2.5 μM) for 18 h. Error bars show SEM for *N* = >3 replicates. ****p < 0.0001.

We previously conducted a cell-based high-throughput screen to identify selective activators of ATF6 signaling.^19^ From this screen, *N*-(2-hydroxy-5-methylphenyl)-3-phenylpropanamide (AA147; Figure 1A) emerged as a selective ATF6 activator that is able to promote adaptive ATF6 activity to mitigate pathologies associated with etiologically diverse disorders.^11,19−26^ We found that AA147 functions as a prodrug, wherein the oxidative conversion of the 2-amino-*p*-cresol substructure by ER-resident oxidases (e.g., cytochrome P450s) leads to an electrophilic quinone methide that covalently engages a subset of ER proteins primarily consisting of protein disulfide isomerases (PDIs).^27^ PDIs maintain ATF6 in disulfide-bonded oligomeric structures that restrain its activation.^28−31^ This suggested that AA147-dependent modification of a subset of PDIs could lead to reduction, monomerization, and subsequent trafficking of ATF6 to the Golgi, where S1/S2-enabled proteolytic cleavage affords the cytosolic ATF6 transcription factor amenable to nuclear localization and transcriptional remodeling. Consistent with this, we showed that genetic depletion of PDIs impaired AA147-dependent ATF6 activation, clearly linking compound modifications of PDIs to the selective ATF6 transcriptional activity observed for this compound.^27^ However, the chemical properties of AA147 connecting PDI modification to the subsequent selective ATF6 activation remain to be fully established.

Intriguingly, another compound identified in our screen, (*E*)-1-(4-fluorophenyl)-3-((2-hydroxy-5-methylphenyl)amino)prop-2-en-1-one (AA132; Figure 1A), also contains the 2-amino-*p*-cresol moiety critical for AA147-dependent ATF6 activation.^27^ Despite containing the same substructure, we found that AA132 showed reduced selectivity for ATF6 transcriptional program activation in comparison to AA147; instead, AA132 globally activates all three UPR signaling pathways.^19^ Understanding the requirements underlying these divergent transcriptional responses would provide insight into pharmacologic ATF6 activation and factors governing UPR activation generally. Furthermore, given the continued demonstration of the beneficial effects of selective pharmacologic ATF6 activators for ameliorating etiologically diverse diseases, such findings could guide medicinal chemistry decisions in designing more potent ATF6-selective proteostasis regulators.

In this study, we address this issue by systematically investigating the activity, selectivity, and mechanism of AA132-dependent UPR activation. We show that AA132 activates UPR signaling through a mechanism similar to that observed for AA147 involving metabolic activation of the 2-amino-*p*-cresol moiety to a putative quinone methide, which then covalently modifies ER proteins, including many PDIs (Figure 1B). However, we demonstrate that AA132 modifies ER-resident PDIs to a greater extent than AA147 using a chemoproteomic probe of AA132. This higher amount of labeling is predicted to cause ER stress, thus activating the global UPR. Consistent with this, we find that administration of AA132 at lower doses that better mimic the level of PDI modification observed with AA147 leads to selective activation of the ATF6 signaling arm of the UPR. This study showcases the dynamic interplay between small chemical scaffold modifications, resulting proteome reactivity, and transcriptional selectivity for metabolically activatable ER proteostasis regulators, providing a framework for the continued development of ATF6 activating compounds for disease intervention.

## Results

### AA132 Activates UPR Signaling through a Mechanism Involving Metabolic Activation and Covalent Protein Modification

We previously reported that AA147 selectively activates ATF6 signaling through a process involving metabolic activation of its 2-amino-*p*-cresol substructure affording a reactive quinone methide that covalently modifies ER-localized PDIs.^27^ AA132 also contains a 2-amino-*p*-cresol moiety, suggesting that this compound could activate UPR signaling through an analogous mechanism (Figure 1A,B). Consistent with this hypothesis, cotreatment of HEK293T cells with an alkyne-modified AA147 analog (AA147^yne^; 10 μM)^27^ and AA132 (40 μM) reduced AA147^yne^ protein labeling, as visualized through appending a Cy5-azide fluorophore to the terminal alkyne of conjugated proteins via Cu(I)-catalyzed azide–alkyne cycloaddition (CuAAC) (Figure 1C).^32,33^ This covalent competition reaction indicates that AA132 engages a similar subset of the proteome as AA147.

Next, we sought to determine the sensitivity of AA132-mediated UPR activation to cotreatment with resveratrol, which blocks metabolic activation, or β-mercaptoethanol (BME), which quenches the activated AA132 quinone methide through direct covalent modification or, more importantly, modulation of cellular reducing capacity (Figure 1B).^34,35^ We confirmed that AA132 activated luciferase-based reporters of ATF6 signaling (ERSE-FLuc),^19^ IRE1 signaling (XBP1-RLuc),^17^ and PERK signaling (ATF4-FLuc), while AA147 only activated the ATF6-selective ERSE-FLuc reporter (Figure S1A–C). This confirms previous results showing that AA132 activates all three signaling arms of the UPR.^19^ Cotreatment with either resveratrol or BME inhibited AA132-dependent activation of all three UPR reporters (Figures 1D,E and S1D–G). These results support a model wherein AA132 activates global UPR signaling through a mechanism involving AA132 ER metabolic activation generating an electrophilic species followed by covalent protein modification (Figure 1B)—a mechanism similar to that observed for selective AA147-dependent ATF6 activation.^27^

### Synthesis and Characterization of an AA132 Affinity-Enrichment Probe to Monitor Protein Modification

**Figure 2:**
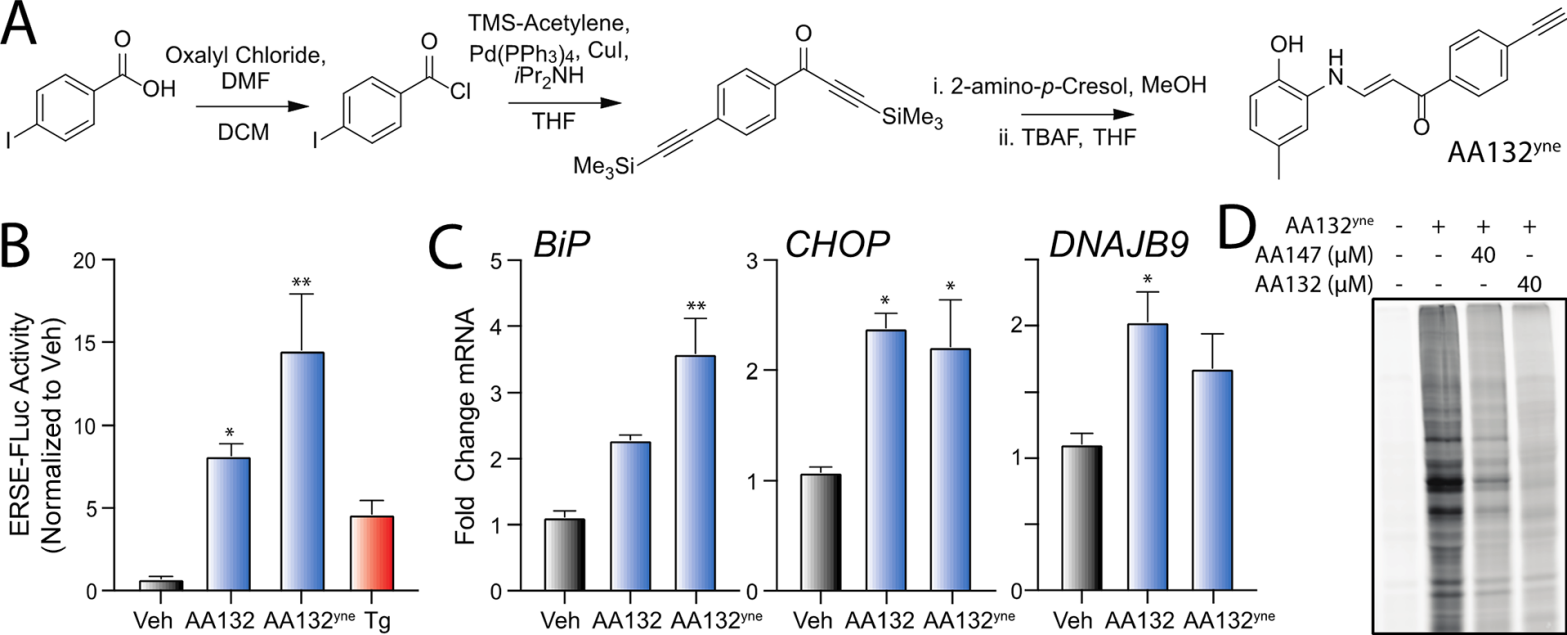
Development of a functional affinity-enrichment probe for AA132. (A) Synthetic scheme for the synthesis of AA132^yne^. (B) Bar graph showing the activation of the ERSE.FLuc ATF6 reporter in HEK293T cells treated with Veh (0.1% DMSO), thapsigargin (Tg; 500 nM), AA132 (10 μM), or AA132^yne^ (10 μM) for 18 h. Error bars show SEM for *n* = 3 replicates. *p < 0.05, **p < 0.01 for one-way ANOVA relative to vehicle-treated cells. (C) Graph showing qPCR of the ATF6 target gene *BiP*, PERK target gene *CHOP*, and XBP1s target gene *DNAJB9* in HEK293T cells treated for 6 h with the indicated compound (10 μM). Error bars show SEM for *N* = 3 biological replicates. *p < 0.05, **p < 0.01 for one-way ANOVA relative to vehicle-treated cells. (D) Representative SDS-PAGE gel of Cy5-conjugated proteins from HEK293T cells treated for 4 h with vehicle (0.1% DMSO), AA132^yne^ (10 μM), the combination of AA132^yne^ (10 μM) and AA147 (40 μM), or the combination of AA132^yne^ (10 μM) and AA132 (40 μM).

Given that AA132 cotreatment reduces AA147^yne^-dependent proteome labeling (Figure 1C), we hypothesized that proteome reactivity differences may underlie the less selective transcriptional response observed with AA132 versus AA147 treatment. To test this hypothesis, we synthesized an AA132 analog where the B-ring *para*-fluorine is replaced with an alkyne to enable subsequent affinity-enrichment experiments (Figure 2A; AA132^yne^). We confirmed that AA132^yne^ activates the ATF6-selective ERSE-FLuc reporter, the IRE1-selective XBP1-RLuc reporter, and the PERK-selective ATF4-FLuc reporter (Figures 2B and S2A,B). Further, AA132^yne^ (10 μM) induced expression of UPR target genes regulated downstream of ATF6 (*HSPA5/BiP*), IRE1 (*DNAJB9*), and PERK (*CHOP/DDIT3*) in HEK293T (Figure 2C) and MEF cells (Figure S2C) at levels similar to those observed with AA132. These results indicate that AA132^yne^, like AA132, globally activates all three arms of the UPR.

We next treated HEK293T cells with AA132^yne^ for 4 h and visualized cellular protein labeling by CuAAC conjugation to a fluorescent azide–cyanine tag followed by sodium dodecyl sulfate-polyacrylamide gel electrophoresis (SDS-PAGE) and in-gel fluorescence scanning (Figure 2D).^36^ Cotreatment with fourfold excess AA147 or AA132 reduces protein labeling by AA132^yne^, providing further evidence that these compounds target similar subsets of the cellular proteome (Figure 2D). As observed with AA147^yne^ (Figure 1C), excess AA132 showed stronger competition with AA132^yne^ labeling than excess AA147. These results establish AA132^yne^ as an efficient probe to monitor protein conjugation by the putative quinone methide reactive species afforded by oxidation of AA132.

### Proteomic Profiling Demonstrates that AA132 Preferentially Modifies ER-Localized Proteins

**Figure 3:**
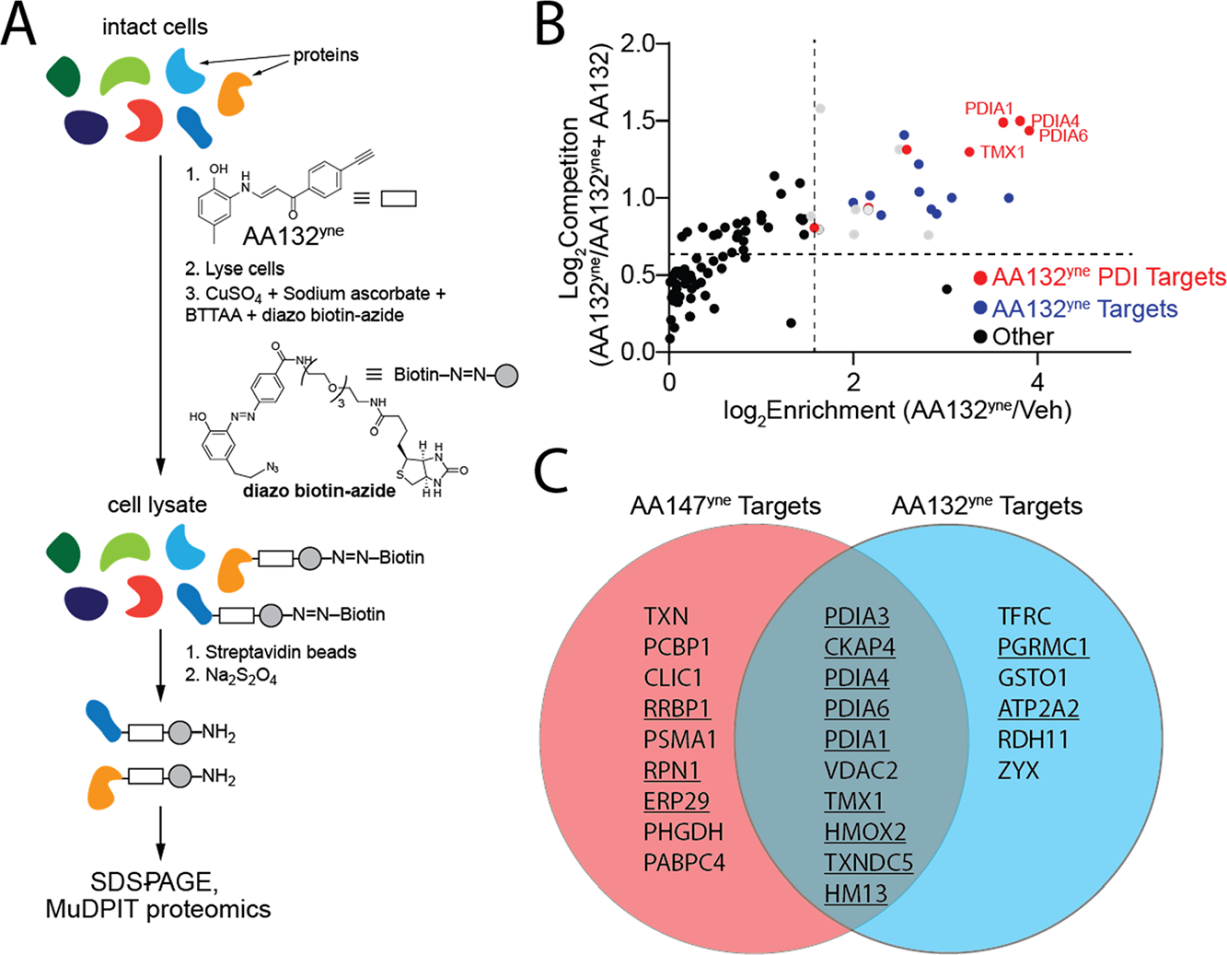
AA132^yne^ covalently modifies ER PDIs. (A) Schematic showing the protocol for affinity purification of proteins covalently modified by AA132^yne^. Reproduced with permission from ref (27). Copyright 2018 *ELIFE*. (B) Plot showing log_2_ fold change of AA132^yne^-enriched proteins relative to vehicle (*x* axis) and log_2_ fold change of competition ratio (AA132^yne^ + Veh/AA132^yne^ + AA132) (*y* axis). Dotted lines indicate significantly enriched proteins (>3-fold; *x* axis) and proteins with a significant competition ratio (>1.5 fold; *y* axis). Red circles identify AA132^yne^ targets with PDI GO annotation (GO:0003756); blue circles identify additional AA132^yne^ targets identified across two separate experiments. Gray circles identify AA132^yne^ targets identified in one experiment. Data are included in Table S1. (C) Venn diagram showing unique targets of AA147^yne^ (red) or AA132^yne^ (blue). Underlined proteins have the GO annotation GO:0044432 “endoplasmic reticulum part”.

Our in-gel fluorescence, SDS-PAGE-based competition experiments using AA147^yne^ and AA132^yne^ suggest that AA132 engages similar targets to a greater extent than AA147, as AA132 competes better for protein labeling than AA147 (Figures 1C and 2D). To scrutinize this hypothesis, we implemented an established affinity-purification mass spectrometry workflow to identify AA132^yne^ protein targets (Figure 3A).^27^ Briefly, we treated HEK293T cells with vehicle, AA132^yne^ (10 μM), or AA132^yne^ (10 μM) with excess AA132 (40 μM) for 6 h. Diazo biotin-azide was then covalently attached to AA132^yne^-conjugated proteins by CuAAC-mediated click chemistry, and the cellular protein conjugates were isolated with streptavidin affinity enrichment. Following tryptic digestion, conjugated proteins were identified by Tandem Mass Tag (TMT)-Multi-Dimensional Protein Identification Technology (MuDPIT) proteomic analysis.^37,38^ AA132^yne^ protein conjugates were further differentiated from non-specific interacting proteins using TMT reporter ion ratios between different conditions. We defined true conjugates by the following criteria: (1) a greater than threefold enrichment ratio from AA132^yne^-treated cells relative to DMSO-treated cells, and (2) a greater than 1.5-fold reduction in enrichment ratio in cells cotreated with AA132^yne^ and excess AA132 relative to cells treated with AA132^yne^ alone (*p* < 0.05) (Figure 3B and Table S1). We identified 16 proteins modified by AA132^yne^, 10 of which were previously identified targets of AA147^yne^ (Figure 3C).^27^ Intriguingly, like AA147^yne^, AA132^yne^ preferentially reacted with ER-localized proteins (11/16, 69% of identified targets are localized to the ER; Figure S3A).^27^ Further, the most abundant members of the PDI family, including PDIA3, PDIA4, PDIA6, PDIA1, and TXNDC5, are well-represented among the shared targets of AA132^yne^ and AA147^yne^. These results indicate that AA132^yne^, like AA147^yne^, selectively modifies ER-resident proteins, most notably PDIs, likely reflecting the similar physicochemical properties between AA147 and AA132 (Figure S3B). Apart from PDIs, AA132^yne^ also labeled the sarco(endo)plasmic reticulum calcium-ATPase 2 (SERCA2/ATP2A2), which utilizes ATP to transport Ca^2+^ into the ER. Pharmacologic inhibition of SERCA using compounds like thapsigargin (Tg) induces global UPR activation through disruptions in ER Ca^2+^, suggesting that AA132-dependent SERCA2 modification could explain the global UPR activation induced by this compound. However, unlike Tg, AA132 did not increase cytosolic Ca^2+^ (Figure S3C), indicating that AA132 is unlikely to induce global UPR activation through this mechanism. Instead, the robust labeling of PDIs by AA132^yne^ supports a model whereby AA132, like AA147,^27^ activates UPR signaling through a mechanism involving covalent modification of ER-localized PDIs (Figure 1B).

### AA132 Shows Enhanced PDI Labeling as Compared to AA147

**Figure 4:**
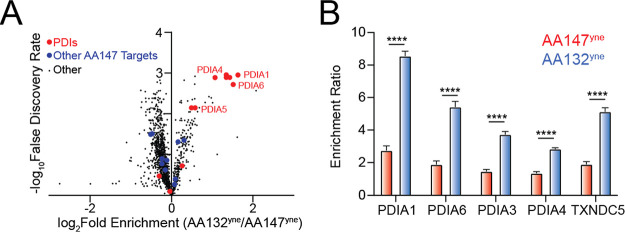
AA132^yne^ shows higher PDI labeling as compared to AA147^yne^. (A) Volcano plot showing log_2_ fold enrichment of AA132^yne^ labeled proteins relative to AA147^yne^ labeled proteins (*x* axis) versus the −log FDR (*y* axis) in HEK293T cells (10 μM, 6 h). Proteins with GO annotation for PDI (GO:0003756) labeled in red and additional previously defined AA147^yne^ targets labeled in blue. Data are shown in Table S2. (B) Bar graph of the TMT reporter ion enrichment ratio of select PDIs from data shown in Figure 4A in HEK293T cells treated with the indicated compound relative to DMSO (*N* = 4 biological replicates). ****p < 0.001 for a two-way ANOVA.

Although AA132 and AA147 label similar ER PDI family members, these two compounds induce distinct transcriptional profiles, with AA147 showing selective ATF6 activation and AA132 showing activation of all three arms of the UPR.^19^ To rationalize the discrepancy between unique transcriptional profiles and engagement of similar cellular targets, we performed quantitative TMT-MuDPIT proteomics to directly compare the relative populations of proteins labeled by AA147^yne^ versus those labeled by AA132^yne^ in HEK293T cells (Table S2). We found that AA132^yne^ and AA147^yne^ enrich for similar proteins (Figure 4A). However, AA132^yne^ showed higher overall labeling of multiple PDIs identified to be involved in AA147-dependent ATF6 activation,^27^ as compared to AA147^yne^ (Figure 4B). These include PDIA1, PDIA3, PDIA4, PDIA6, and TXNDC5 (Figure 4A,B). Similar results were observed in other cell types, including liver-derived HepG2 cells (Figure S4A,B and Table S2). We confirmed the increased labeling of PDIA1, PDIA3, PDIA4, and PDIA6 in HEK293T cells by biotin conjugation, streptavidin enrichment, and quantitative immunoblotting of eluted proteins (Figure S4C–F). These results suggest that increased modification of PDIs by AA132 could define the differential transcriptional signaling observed between AA147 and AA132.

### PDI Labeling by AA132^yne^ Approaches Completion More Slowly than PDI Labeling by AA147^yne^

Given the close structural homology between the AA147 and AA132 scaffolds, we sought to identify factors potentially mediating the divergence in proteome labeling and transcriptional selectivity between the two compounds. We initially predicted that small molecule conjugation to target proteins may result in ligand-induced changes in thermal stability. As AA132 and AA147 differ structurally in the linker region, we hypothesized that this small chemical change might lead to a differential effect on thermal stability of conjugated proteins. However, we did not see significant differences in resistance to heat denaturation for PDIA1 upon conjugation with AA147 or AA132 (Figure S5A,B). An alternate hypothesis is that small molecule conjugation to a protein of interest could lead to protein destabilization and subsequent degradation. However, we did not observe changes in levels of PDIA1 upon compound treatment, as assessed by quantitative immunoblotting (Figure S5C,D).

**Figure 5:**
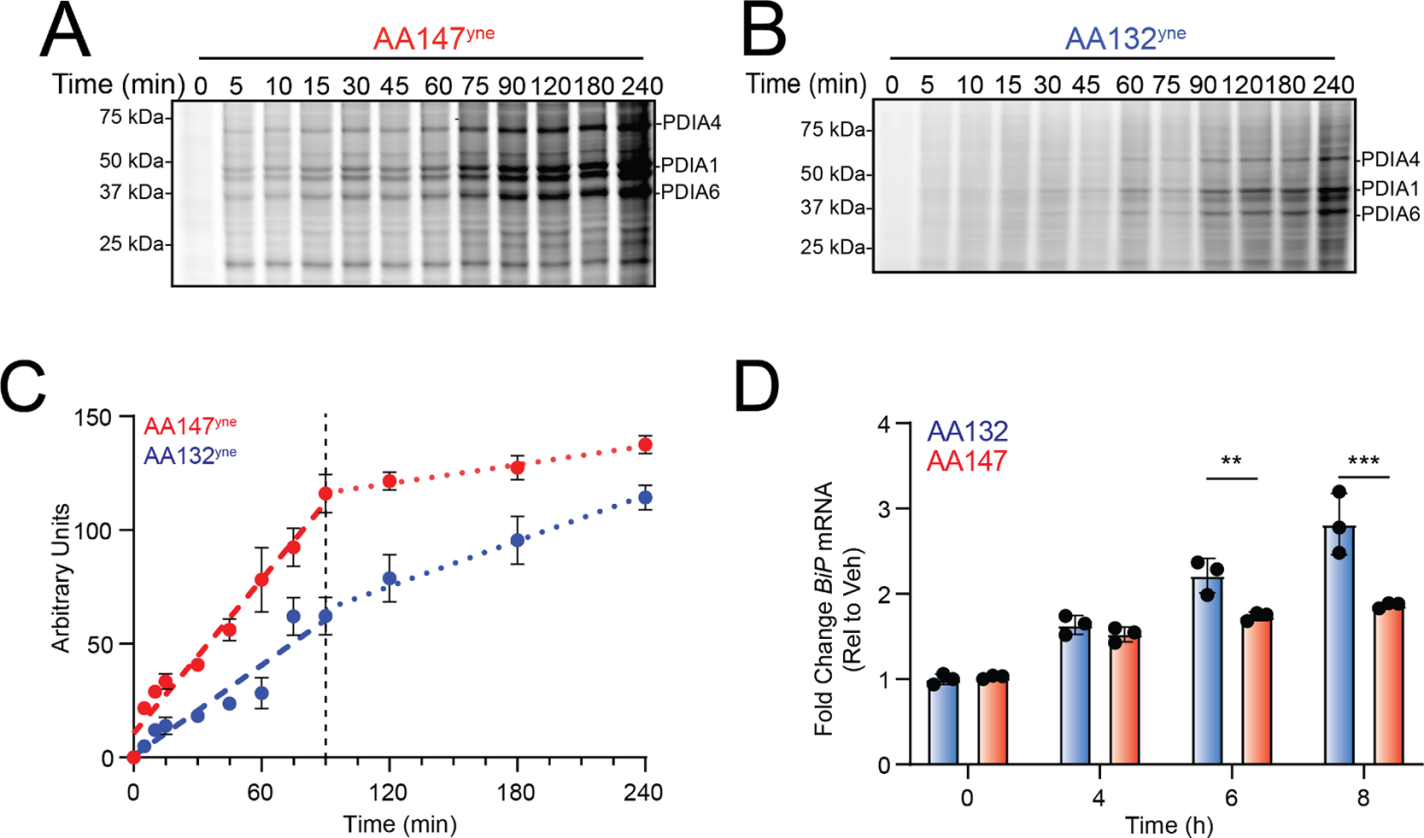
AA132^yne^ shows slower protein labeling kinetics as compared to AA147^yne^. (A) Representative SDS-PAGE gel of Cy5-conjugated proteins from HEK293T cells treated for the indicated time point with AA147^yne^ (10 μM). (B) Representative SDS-PAGE gel of Cy5-conjugated proteins from HEK293T cells treated for the indicated time point with AA132^yne^ (10 μM). (C) Quantification of gels described in Figure 5A,B. The *y* axis is lane intensity at each time point (arbitrary units). Error bars represent SEM (*N* = 4 biological replicates). The slopes of the curves as the PDI labeling reaction progressed were quantified using linear regression on the data points from 0 to 90 min, and from 90 to 240 min (i.e., before and after the dashed vertical line). The ratios of these slopes were 0.15 ± 0.08 for AA147^yne^ and 0.55 ± 0.14 for AA132^yne^ (mean ± standard error, *N* = 4). Thus, the slope diminishes significantly more for AA147^yne^ than for AA132^yne^ (*p* = 0.039, two-tailed *t*-test), indicating that the reaction is closer to its plateau for AA147^yne^. (D) Graph showing qPCR of the ATF6 target gene *BiP* for HEK293T cells treated with AA147 (10 μM) or AA132 (10 μM) at the indicated time points. Error bars represent SEM (*N* = 3 biological replicates). **p < 0.01, ***p < 0.005 for two-way ANOVA.

We next tested whether differences in the relative rates of conjugate formation by AA147 or AA132 could explain the differential proteome labeling between AA147 and AA132. We performed an in-gel fluorescence time course experiment in HEK293T cells monitoring AA147^yne^ or AA132^yne^ conjugate formation (Figure 5A–C). Interestingly, these results show that PDI modification by AA147^yne^ is closer to completion at the end of this 6 h time course, as compared to PDI modification by AA132^yne^. This is evident from the ratio of the slope of AA147^yne^-modified PDI band intensity from 90–240 min to the slope observed from 0–90 min being 0.15 ± 0.07 (mean ± standard error, *N* = 4), consistent with this curve approaching a plateau. In contrast, this ratio is significantly higher for AA132^yne^ (0.55 ± 0.14, mean ± standard error, *N* = 4; *p* = 0.039, two-tailed *t*-test), indicating that modification of PDIs by this compound is further from plateauing. Similar results were observed in other cell types, including ALMC2 plasma cells (Figure S5E,F). Thus, it appears that the PDI modification activity of AA147^yne^ is exhausted faster than that of AA132^yne^. This could be either because the ER oxidase that converts these compounds to their active forms becomes deactivated, or because AA147^yne^ depletes the targets available to it—that is, those that are close enough to the site of oxidation that activated AA147^yne^ can diffuse to them before it is quenched—faster than AA132^yne^ does.

These results, in the context of the small structural differences in the linker region, suggest differences in the nature of the metabolically generated electrophilic species between AA147 and AA132. Such differences could lead to a strong nucleophile dependence on the quenching susceptibility of activated AA147 vs AA132. For example, *o*-quinone methide is 80-fold more reactive with water than *p*-quinone methide, although their reactivities with thiocyanate are similar.^42,43^ To investigate the quenching susceptibility of the activated electrophiles, we treated HEK293T cells with AA132^yne^ or AA147^yne^ in the presence or absence of BME. We found that cotreatment with BME showed greater inhibition of AA132^yne^ labeling, as compared to AA147^yne^ labeling (Figure S5G,H). This suggests that activated AA147^yne^ is less sensitive to increases of intracellular reduction potential, as compared to AA132^yne^.

Based on our model, one would expect the slower plateauing of PDI modification by AA132 relative to AA147 to be reflected in the extent of UPR activation. To test this, we monitored the induction of the ATF6 target gene *BiP* in HEK293T cells treated with AA147 or AA132 for 4, 6, or 8 h (Figure 5D). As expected, AA132 induced *BiP* to a higher level than AA147 and this level was still increasing robustly at the 8 h timepoint, whereas *BiP* induction by AA147 increased only modestly after 4 h. Together, these results indicate that the higher PDI labeling activity by AA132 extends and increases the activation of UPR signaling. This is consistent with a model wherein activated AA132 either has a longer intracellular lifetime or is produced at a different level than AA147, allowing this compound to label PDIs to a greater extent and subsequently induce global UPR activation.

### Dose-Dependent Regulation of AA132 Transcriptional Selectivity

**Figure 6:**
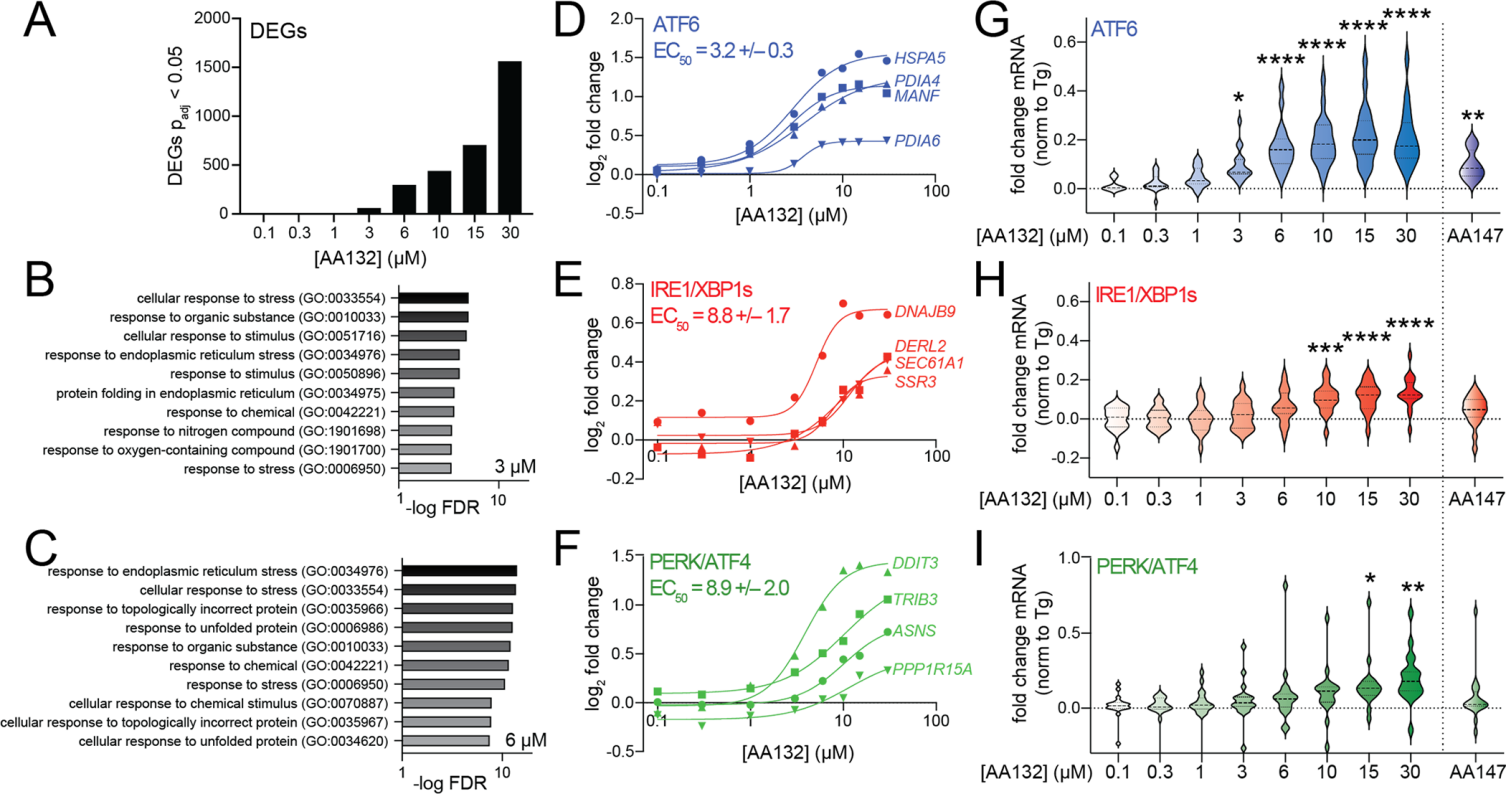
AA132 selectively activates ATF6 transcriptional signaling at lower doses. (A) Differentially expressed genes (*p*_adj_ < 0.05) from RNAseq data of HEK293T cells treated with vehicle or the indicated dose of AA132 for 6 h (*N* = 3 replicates per condition). RNAseq data are included in Table S3. (B, C) Top-10 GO terms for significantly induced genes (fold change > 1.3, *p*_adj_ < 0.05) identified by RNAseq in HEK293T cells treated with 3 μM (B) or 6 μM (C) AA132 for 6 h. RNAseq data are included in Table S3. Full GO analysis is included in Table S4. (D–F) Fold change, relative to the vehicle, for select ATF6 target genes (D), IRE1/XBP1s target genes (E), or PERK/ATF4 target genes (F) from RNAseq data of HEK293T cells treated with increasing concentrations of AA132 for 6 h. The average EC_50_ for the four target genes representing each UPR pathway is shown. (G–I) Fold change, relative to the vehicle, for genesets of 15–20 genes regulated downstream of ATF6 (G), IRE1/XBP1s (H), or PERK/ATF4 (I) from RNAseq of HEK293T cells treated with increasing concentrations of AA132 for 6 h. The fold change expression of individual genes was normalized to that observed with the global ER stressor thapsigargin (Tg), as reported previously.^19,44^ The impact of AA147 (10 μM; 6 h) on these genesets is shown on the right. The expression of UPR genesets is shown in Table S5. *p < 0.05, **p < 0.01, ***p < 0.005, ****p < 0.001 for one-way ANOVA.

AA132 and AA147 induce distinct transcriptional profiles in HEK293T cells after 6 h treatment. AA147 selectively activates the ATF6 arm of the UPR, while AA132 activates all three arms of the UPR.^19^ Our results indicate that the distinct transcriptional profiles induced by these two compounds could be attributed to differences in PDI labeling, with AA132 modifying PDIs to a greater extent than AA147 (Figure 4A,B). This would suggest that decreasing AA132-dependent protein modification should result in increased transcriptional selectivity for ATF6 activation. To test this, we monitored mRNA expression by RNAseq in HEK293T cells treated with increasing concentrations of AA132 from 0.1–30 μM for 6 h (Table S3). We confirmed dose-dependent protein modification in HEK293T cells treated with increasing concentrations of AA132 (Figure S6A). As expected, the number of differentially expressed genes increased with increasing AA132 concentrations (Figure 6A). Gene ontology (GO) analysis of significantly induced genes in AA132-treated cells showed enrichment in terms associated with ER function, ER stress, and the UPR (Figures 6B,C, S6B–D, and Table S4). These results confirm the genome-wide transcriptional specificity of AA132 for UPR activation reported previously.^19^

Next, we sought to define the transcriptional selectivity of increasing concentrations of AA132 for activation of the three arms of the UPR. Monitoring expression of select target genes of ATF6 (*HSPA5*, *PDIA4*, *PDIA6*, *MANF*), IRE1/XBP1s (*DNAJB9*, *SSR3*, *SEC61A*, *DERL2*), and PERK/ATF4 (*DDIT3*, *PPP1R15A*, *ASNS*, *TRIB3*) shows dose-dependent increases of these genes (Figure 6D–F). Interestingly, the EC_50_ for ATF6-target genes (3.2 μM) is less than that observed for IRE1/XBP1s and PERK/ATF4 target genes (8.8 and 8.9 μM, respectively). This suggests that ATF6 signaling is induced at lower concentrations of AA132, as compared to IRE1/XBP1s and PERK/ATF4. Further, normalizing the expression of 15–20 target genes regulated by ATF6, IRE1/XBP1s, or PERK/ATF4 observed following 6 h treatment with AA132 to that observed with the global ER stressor thapsigargin (Tg) shows that ATF6 target genes are significantly induced starting at 3 μM, while IRE1/XBP1s and PERK/ATF4 target genesets are induced at 10 and 15 μM, respectively (Figure 6G–I and Table S5).^44^ Interestingly, the activation of these genesets observed at 3 μM AA132 matches the selective ATF6 activation observed in HEK293T cells treated with AA147 (10 μM, 6 h; Figure 6G–I, right). These results support a model wherein lower concentrations of AA132 (3–6 μM) preferentially activate ATF6 transcriptional signaling, while higher concentrations (>10 μM) lead to global UPR activation.

## Discussion

Here, we sought to define the molecular basis for the differential transcriptional selectivity of AA147 and AA132 in the context of arm-selective UPR activation. Like AA147, we show that AA132 achieves pharmacologic UPR modulation through a mechanism involving metabolic oxidation of its 2-amino-*p*-cresol moiety, yielding a putative quinone methide that subsequently covalently modifies ER-localized proteins, most notably multiple PDIs.^27^ Despite sharing numerous protein targets, comparative chemoproteomic experiments revealed that AA132 labels several PDIs to a greater extent than AA147 at treatment times exceeding 4 h. This divergence in relative levels of ER proteome labeling provides a mechanism to explain the differential selectivity for UPR activation observed upon AA132 or AA147 treatment, with AA132 activating all three arms of the UPR and AA147 selectively activating ATF6 UPR signaling.^19^ Our results indicate that this greater protein labeling could result from either increased production or greater reactivity toward PDIs relative to its reactivity with quenchers for metabolically activated AA132 than activated AA147. This would allow access to a larger pool of PDIs. Thus, the increased labeling of PDIs afforded by AA132 provides a mechanism to explain the global activation of UPR signaling pathways observed with this compound, as higher labeling could lead to disruptions in the basal activities of PDIs and, subsequent, UPR activation. In contrast, the more modest PDI inhibition induced by AA147 selectively promotes ATF6 reduction, monomerization, and trafficking, leading to selective activation of ATF6 transcriptional activity without larger disruptions of PDI function. Consistent with this, lower concentrations of AA132 decrease protein modifications and lead to selective ATF6 activation, mimicking the transcriptional selectivity observed with AA147. Together, these results highlight the connection between electrophilic reactivity and transcriptional selectivity for pharmacologic ATF6 activators, establishing new opportunities to develop next-generation compounds of this class with improved activity and selectivity.

While we and others have demonstrated the importance of PDI family members in dictating ATF6 activity, the PDIs also have critical roles in regulating the IRE1 and PERK arms of the UPR. PDIA6 interacts with luminal domains of both PERK and IRE1 to prevent hyperactivation of these UPR pathways.^45^ Similarly, both PDIA1 and PDIA3 are implicated in regulating PERK signaling in cancer cells in the presence or absence of ER stress.^46^ In addition, other PDIs, including PDIA5 and ERP18, interact with ATF6 luminal domain disulfides to regulate ATF6 anterograde trafficking to the Golgi prior to proteolytic activation.^28−31^ Previous results indicate that AA147 activates ATF6 signaling through selective modification of only a subset of specific ER PDIs.^27^ For example, AA147 was shown to modify only ∼20% of PDIA4 within the ER.^27^ This suggests that the ability of AA147 to selectively activate ATF6 signaling lies in its unique capacity to modify a small subpopulation of specific PDIs. Consistent with this, genetic depletion of PDIs, including *PDIA1*, *PDIA3*, *PDIA4*, and *PDIA5*, limit AA147-dependent ATF6 activation.^27^

Our results suggest that the increased modification of PDIs afforded by AA132 underlies its ability to globally activate UPR signaling. We show that AA132 modifies multiple PDIs to a greater extent than AA147. This could lead to increased disruptions in ER function that induce global UPR activation. Alternatively, increased modifications of certain PDIs such as PDIA1 or PDIA6 could directly influence the activity of UPR signaling pathways. Highly selective covalent PDIA1 inhibitors that modify PDIA1 to greater extents than AA147 can activate IRE1/XBP1s signaling.^47,48^ This suggests that increased targeting of PDIA1 by AA132 may contribute to the IRE1/XBP1s activation observed under these conditions. Similarly, increased PDIA6 modification could mitigate the repression of IRE1 and PERK hyperactivity afforded by this PDI,^45^ increasing activity of these UPR signaling pathways. Both these models predict that reducing modifications of PDIs should decrease IRE1 and PERK signaling and allow for selective ATF6 activation. Consistent with this, we show that decreasing AA132-dependent modifications of PDIs, including of PDIA1 and PDIA6, by reducing its concentration decreases activation of IRE1 and PERK transcriptional signaling and leads to preferential ATF6 transcriptional activity. This suggests that global UPR activation afforded by AA132 can be viewed as a graded disruption in the tightly controlled regulatory roles of ER PDIs involved in regulating ER proteostasis and UPR signaling.

AA132 and AA147 have close structural similarity, with both compounds containing the 2-amino-*p*-cresol substructure critical for the metabolic activation needed for compound activity. However, AA132 contains an enaminone linkage, while AA147 contains an amide linkage, and this subtle modification in the linker region is apparently critical to the activity of these compounds as ER stress pathway activators. Differences in affinities for the requisite metabolic enzyme, contrasting production of ineffective metabolites (i.e., metabolites not directly involved in activating the UPR), differential CYP450 inhibition propensities, and differences in the reactivity of the electrophiles derived from AA147 and AA132 could all be responsible for our observations of slower kinetics of proteome labeling at early time points but a greater eventual extent of PDI reactivity at later time points for the enaminone class of ER proteostasis regulators.

Our chemoproteomic results provide insight into the narrow window of PDI conjugation necessary for selective ATF6 activation mediated by metabolically activatable ER proteostasis regulators. In this view, the activity of a few of the PDIs can be viewed as a rheostat controlling a spectrum of UPR transcriptional activities. From a therapeutic perspective, concomitant activation of ATF6 and IRE1 signaling leads to a uniquely remodeled ER proteostasis environment preferable for some disease contexts, as compared to activation of individual UPR pathways.^13,62^ Thus, the extent of global UPR activation observed with AA132 may be of some value in certain disease contexts. Regardless, a better understanding of the factors driving selectivity of the transcriptional response induced by ER proteostasis regulators is essential for the development of improved ATF6 activators for the treatment of etiologically diverse diseases.

## Materials and Methods

### Cell Culture

HEK293T-Rex (ATCC), HEK293T (ATCC), and HepG2 (ATCC) were cultured in high-glucose Dulbecco’s modified Eagle’s medium supplemented with glutamine, penicillin/streptomycin, and 10% fetal bovine serum (FBS). Cells were routinely tested for mycoplasma every 6 months. We did not further authenticate the cell lines. ALMC-2 cells (a kind gift from Diane Jelinak’s laboratory) were cultured in Iscove’s modified Dulbecco’s medium GlutaMAX (Life Technologies) supplemented with penicillin/streptomycin, 5% FBS, and 2 ng/mL interleukin-6 (IL-6). All cells were cultured under typical tissue culture conditions (37 °C, 5% CO_2_).

### Measurement of UPR Activity Using Luciferase Reporters

HEK293T-Rex cells expressing the ERSE.FLuc,^19^ XBP1s.RLuc,^19^ or ATF4.FLuc reporter were plated at 100 μL/well from suspensions of 200,000 cells/mL in white clear-bottom 96-well plates (Corning) and incubated at 37 °C overnight. The following day, cells were treated with 25 μL of compound-containing media to give the final concentration as described before incubating for 18 h at 37 °C. The plates were equilibrated to room temperature, then either 125 μL of Firefly luciferase assay reagent-1 (ERSE.FLuc and ATF4.FLuc) or Renilla luciferase assay reagent-1 (XBP1s.RLuc) (Targeting Systems) were added to each well. Samples were dark adapted for 10 min to stabilize signals. Luminescence was then measured in an Infinite F200 PRO plate reader (Tecan) and corrected for the background signal (integration time 250 ms). All measurements were performed in biological triplicate.

### Quantitative RT-PCR

The relative mRNA expression levels of target genes were measured using quantitative reverse transcription polymerase chain reaction (RT-PCR). Cells were treated as described at 37 °C, harvested by trypsinization, washed with Dulbecco’s phosphate-buffered saline (GIBCO), and then RNA was extracted using the QuickRNA Miniprep kit (Zymo). qPCR reactions were performed on cDNA prepared from 500 ng of total cellular RNA using the high-capacity cDNA reverse transcription kit (Applied Biosystems). PowerSYBR green PCR master mix (Applied Biosystems), cDNA, and appropriate primers purchased from Integrated DNA Technologies (see Table 1) were used for amplifications (6 min at 95 °C, then 45 cycles of 10 s at 95 °C, 30 s at 60 °C) in an ABI 7900HT fast real-time PCR machine. Primer integrity was assessed by a thermal melt to confirm homogeneity and the absence of primer dimers. Transcripts were normalized to the housekeeping genes RPLP2, and all measurements were performed in biological triplicate. Data were analyzed using the RQ Manager and DataAssist 2.0 software (ABI). qPCR data are reported as mean ± standard error plotted using Prism GraphPad.

**Table 1:** Sequences of Primers for qPCR

gene	forward primer	reverse primer
*HSPA5/BiP*	GCCTGTATTTCTAGACCTGCC	TTCATCTTGCCAGCCAGTTG
*DNAJB9/ERDJ4*	GGAAGGAGGAGCGCTAGGTC	ATCCTGCACCCTCCGACTAC
*HERPUD1*	AACGGCATGTTTTGCATCTG	GGGGAAGAAAGGTTCCGAAG
*PDIA4*	AGTGGGGAGGATGTCAATGC	TGGCTGGGATTTGATGACTG
*SEC24D*	AGCAGACTGTCCTGGGAAGC	TTTGTTTGGGGCTGGAAAAG
*SEL1L*	ATCTCCAAAAGGCAGCAAGC	TGGGAGAGCCTTCCTCAGTC
*DDIT3/CHOP*	ACCAAGGGAGAACCAGGAAACG	TCACCATTCGGTCAATCAGAGC
*RPLP2*	CGTCGCCTCCTACCTGCT	CATTCAGCTCACTGATAACCTTG

### SDS-PAGE In-Gel Fluorescence Scanning

ALMC-2 cells (200,000 cells/mL) or HEK293T cells (250,000 cells/well) were treated with the indicated compound in six-well plates at indicated concentration and time period. Cells were lysed in radioimmunoprecipitation assay (RIPA) buffer (150 mM NaCl, 50 mM Tris pH 7.5, 1% Triton X-100, 0.5% sodium deoxycholate, and 0.1% SDS) supplemented with fresh protease inhibitor cocktail (Roche, Indianapolis, IN) and centrifuged for 20 min at 16,000 × *g* following a 30 min incubation. Protein concentration of the supernatant was determined by the BCA assay (Thermo Fisher) and normalized to give 42.5 μL at 2.35 mg/mL (100 μg/total protein). ‘Click chemistry master mix’ (7.5 μL) was added to each sample to give final concentrations of 100 μM of Cy5-azide (Click Chemistry Tools, Scottsdale, AZ), 800 μM copper(II) sulfate, 1.6 mM BTTAA ligand (2-(4-((bis((1-*tert*-butyl-1*H*-1,2,3-triazol-4-yl)methyl)amino)methyl)-1*H*-1,2,3-triazol-1-yl)acetic acid) (Albert Einstein College), and 5 mM sodium ascorbate. Reaction incubated at 30 ° C for 1 h while shaking before CHCl_3_/MeOH protein precipitation. Dried protein was redissolved in 20 μL 1× SDS loading buffer and 25 μg of it was loaded on gel for SDS-PAGE in-gel fluorescence scanning and subsequently visualized using an Odyssey Infrared Imaging System (Li-Cor Biosciences).

### Immunoblotting of AA132^yne^ or AA147^yne^ Conjugated Proteins

HEK293T cells grown to 80–90% confluency in 10 cm plates were treated with 10 μM indicated compound (AA147^yne^, AA132^yne^, or Veh) for 6 h at 37 °C. The cells were washed with PBS before harvesting with trypsin, pelleting (500 g, 5 min), and washed with PBS (1 mL). Cell pellets were resuspended in RIPA buffer before sonication with a probe tip sonicator to lyse the cells (15 cycles, 3 s on/2 off, 30% amplitude). The lysates were cleared via centrifugation and the concentration of protein was adjusted to 4 mg/mL using the BCA assay. 2 g protein (500 μL) was taken and reacted with a mixture of diazo biotin-azide (100 μM), copper(II) sulfate (800 μM), BTTAA (1.6 mM), sodium ascorbate (5.0 mM) for 90 min at 30 °C with shaking (600 rpm). The reaction was quenched with the sequential addition of cold methanol (4× volume), chloroform (1× volume), and DPBS (4× volume) to precipitate proteins. Proteins were pelleted by centrifugation (4700 g, 10 min, 4 °C). The supernatant was discarded, and the pellets were dried under air for 5 min. Protein pellets were resuspended in 6 M urea in PBS (500 μL) with brief sonication. A sample (25 μL) was taken as the “input” for western blot analysis. The sample diluted with 5.5 mL DPBS (0.2% SDS) and streptavidin agarose resin (100 μL, washed 3 × 1 mL with PBS) was added to each sample before incubation for 18 h at 24 °C with rotation. The beads were pelleted via centrifugation (3000*g*, 2 min) and washed with 0.2% SDS in DPBS, DPBS (2 × 2 mL). 100 μL freshly prepared 50 mM sodium dithionite was added to beads to cleave protein conjugates and incubated at 30 ° C for 1 h. Supernatant transferred to new 2 mL eppendorf and precipitated with cold methanol (4× volume), chloroform (1× volume), and DPBS (4× volume). Proteins resuspended in 1× SDS-PAGE gel loading buffer (20 μL) and heated for 5 min at 95 °C prior to separation via SDS-PAGE and transfer to poly(vinylidene difluoride) membranes for immunoblot analysis of indicated proteins (rabbit PDIA4 (1:2000) (Protein Tech), rabbit PDIA3 (1:1000) (Protein Tech), mouse GAPDH (1:1000) (Cell Signaling), goat anti-rabbit IRdye 800-cw (Licor) (1:10,000), and goat anti-mouse IRdye-680RD (Licor) (1:10,000).

### RNA-seq

Cells were lysed and total RNA was collected using the QuickRNA Miniprep kit from Zymo Research (R1055) according to manufacturer’s instructions. RNA concentration was then quantified by NanoDrop. Whole transcriptome RNA was then prepared and sequenced by BGI Americas on the BGI Proprietary platform, which provided paired-end 50 bp reads at 20 million reads per sample. Each condition was performed in triplicate. RNAseq reads were aligned using DNAstar Lasergene SeqManPro to the Homo_sapiens-GRCh38.p7 human genome reference assembly, and assembly data were imported into ArrayStar 12.2 with QSeq (DNAStar Inc.) to quantify the gene expression levels and normalization to reads per kilobase per million. Differential expression analysis was assessed using DESeq2 in R, which also calculated statistical significance calculations of treated cells compared to vehicle-treated cells using a standard negative binomial fit of the reads per kilobase per million data to generate fold-change quantifications. GO analysis was performed using Panther (geneontology.org).^65,66^ The complete RNA-seq data are deposited in gene expression omnibus as GSE227126.

#### Statistical Analysis

Unless otherwise noted, the data were tested for significance using one-way ANOVA with a post hoc Dunnett’s test.

Additional Materials and Methods are included in Supporting Information.

## Supporting Information Available

Contains supplemental materials and methods, Figures S1–S6, Supplementary Tables S1–S4, additional synthetic procedures, and characterization data for synthetic compounds (PDF)
Table S1. Enrichment and competation ratios for proteins identified as targets of AA132^yne^ (XLSX)
Table S2. Fold enrichment for AA132^yne^/AA147^yne^ labeled proteins in HEK293T and HepG2 cells (XLSX)
Table S3. DESEQ outputs for HEK293T cells treated with AA132 or AA147 (XLSX)
Table S4. GO analysis for RNASeq data of HEK293T cells treated with AA132 (XLSX)
Table S5. Expression of ATF6, IRE1/XBP1s, and PERK transcriptional targets in HEK293T cells treated with AA132 or AA147 (XLSX)
