# Supplementary material for: Divergent Proteome Reactivity Influences Arm-Selective Activation of the Unfolded Protein Response by Pharmacological Endoplasmic Reticulum Proteostasis Regulators
Source: ACS Chem Biol. 2023 Jul 31;18(8):1719–29. doi: 10.1021/acschembio.3c00042 (PMC10442855; doi:10.1021/acschembio.3c00042)

Supporting Information

Divergent Proteome Reactivity Influences Arm-Selective Activation of the Unfolded Protein Response by Pharmacologic Endoplasmic Reticulum Proteostasis Regulators

Gabriel M. Kline[1,^], Ryan J Paxman[1,^], Chung-Yon Lin[1], Nicole Madrazo[2], Leonard Yoon[1], Julia M. Grandjean[2], Kyunga Lee[1], Karina Nugroho[1], Evan T. Powers[1], R. Luke Wiseman[2,*] , Jeffery W. Kelly[1,3,*]

[1]Department of Chemistry, The Scripps Research Institute, La Jolla, CA 92037, USA
[2]Department of Molecular Medicine, The Scripps Research Institute, La Jolla, CA 92037, USA
[3]The Skaggs Institute for Chemical Biology, The Scripps Research Institute, La Jolla, CA, 92037, USA
^These authors contributed equally.

*To whom correspondence should be addressed:

Jeffery W. Kelly
Department of Chemistry
The Scripps Research Institute
La Jolla, CA, USA, 92037
Email: jkelly@scripps.edu

R. Luke Wiseman
Department of Molecular Medicine
The Scripps Research Institute
La Jolla, CA, USA, 92037
Email: wiseman@scripps.edu

KEYWORD: unfolded protein response (UPR); ATF6; protein disulfide isomerase (PDI); covalent protein modification; endoplasmic reticulum proteostasis

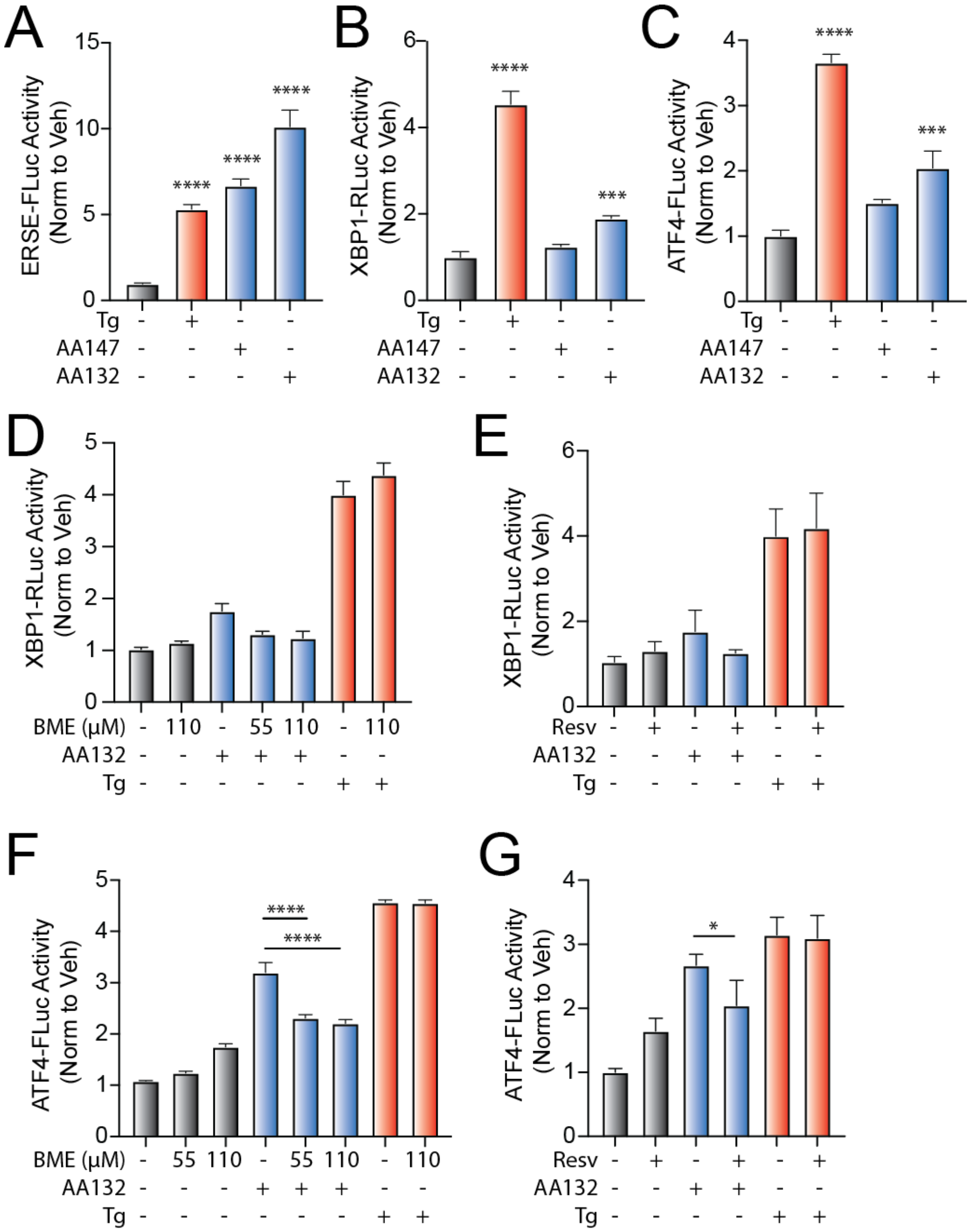

Figure S1. AA132 activates ATF6 signaling pathways through a mechanism involving metabolic activation and covalent protein modification. A-C. Bar graph showing the activation of the ERSE.FLuc ATF6 reporter (**A**), the XBP1-RLuc IRE1 reporter (**B**), or the ATF4-FLuc PERK reporter (**C**) in HEK293T cells treated with Veh (0.1% DMSO), thapsigargin (Tg; 500 nM), AA147 (10 μM), or AA132 (10 μM) for 18 hr. Error bars show SEM for 6 (**A**) or 3 (**B**,**C**) independent replicates. *p<0.05, ***p<0.005, ****p < 0.0001 for one-way ANOVA relative to vehicle-treated cells. **D.** Bar graph showing the activation of the XBP1s.RLuc IRE1 reporter in HEK293T cells treated with AA132 (10 μM) or Tg (500 nM) in the presence or absence of β-mercaptoethanol (BME; 55 μM or 110 μM) for 18 hr. Error bars show SEM for >6 independent replicates**. E.** Bar graph showing the activation of the XBP1s.RLuc IRE1 reporter in HEK293T cells treated with AA132 (10 μM) or Tg (500 nM) in the presence or absence of resveratrol (2.5 μM) for 18 hr. Error bars show SEM for >6 independent replicates. **F.** Bar graph showing the activation of the ATF4.FLuc PERK reporter in HEK293T cells treated with AA132 (10 μM) or Tg (500 nM) in the presence or absence of β-mercaptoethanol (BME; 55 μM or 110 μM) for 18 hr. Error bars show SEM for >6 independent replicates. ****p < 0.0001 for one-way ANOVA. **G.** Bar graph showing the activation of the ATF4.FLuc PERK reporter in HEK293T cells treated with AA132 (10 μM) or Tg (500 nM) in the presence or absence of resveratrol (2.5 μM) for 18 hr. Error bars show SEM for 5 independent replicates. *p < 0.05 for one-way ANOVA.

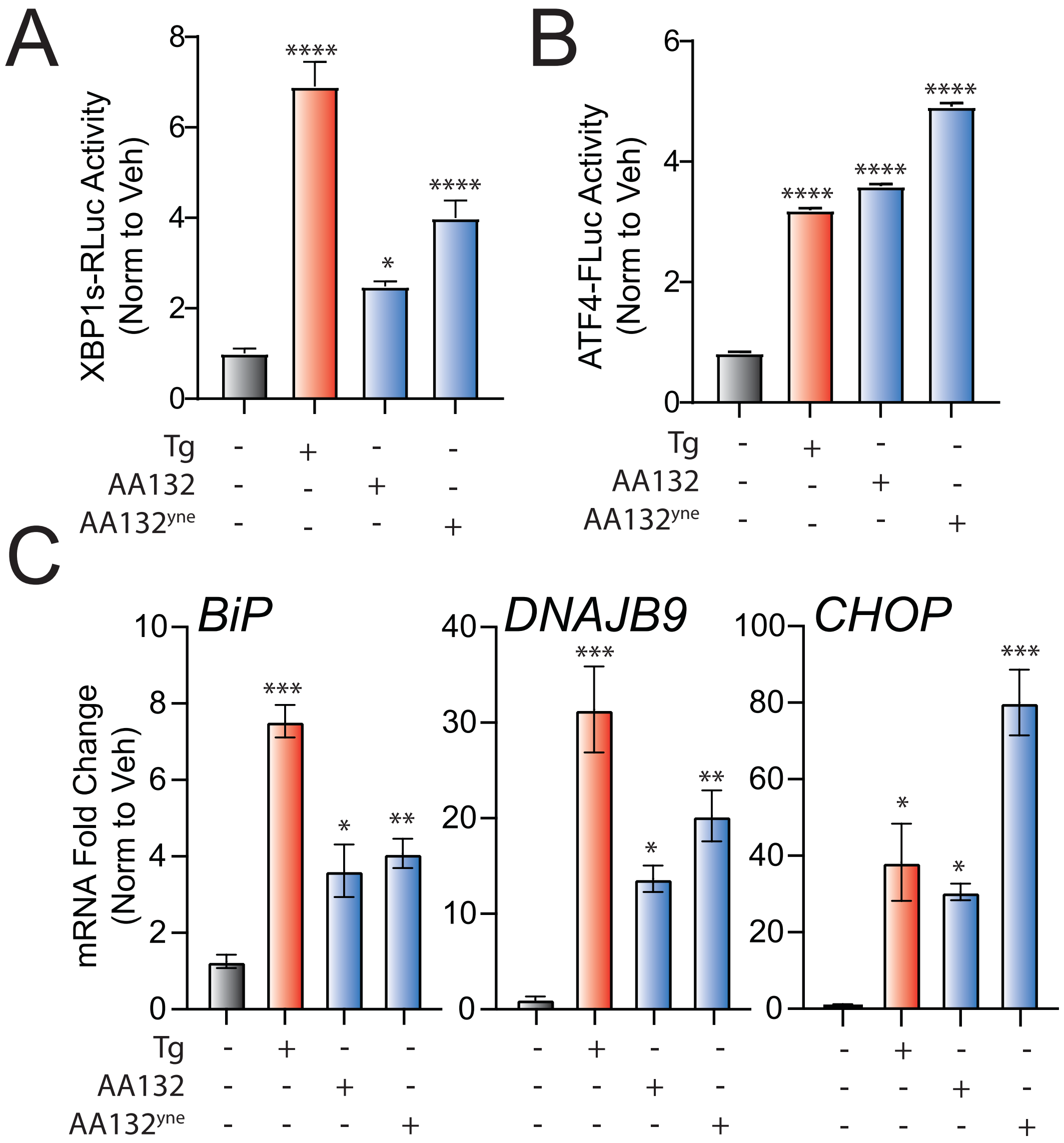

Figure S2. Development of a Functional Affinity Enrichment Probe for AA132. A,B. Bar graph showing the activation of the XBP1s.RLuc IRE1 reporter (**A**) or the ATF4.FLuc PERK reporter (**B**) in HEK293T cells treated with Veh (0.1% DMSO), thapsigargin (Tg; 500 nM), AA132 (10 μM), or AA132^{yne} (10 μM) for 18 hr.Error bars show n= 5 (**A**) or 6 (**B**) independent replicates. *p<0.05, ****p<0.001 for one-way ANOVA. **C.** Graph showing qPCR of the ATF6 target gene *BiP*, PERK target gene *CHOP*, and XBP1s target gene *DNAJB9* in MEF cells treated for 6 h with the indicated compound (10 μM). Error bars show SEM for n = 3 biological replicates. *p<0.05, **p<0.01, ***p<0.001 for one-way ANOVA. .

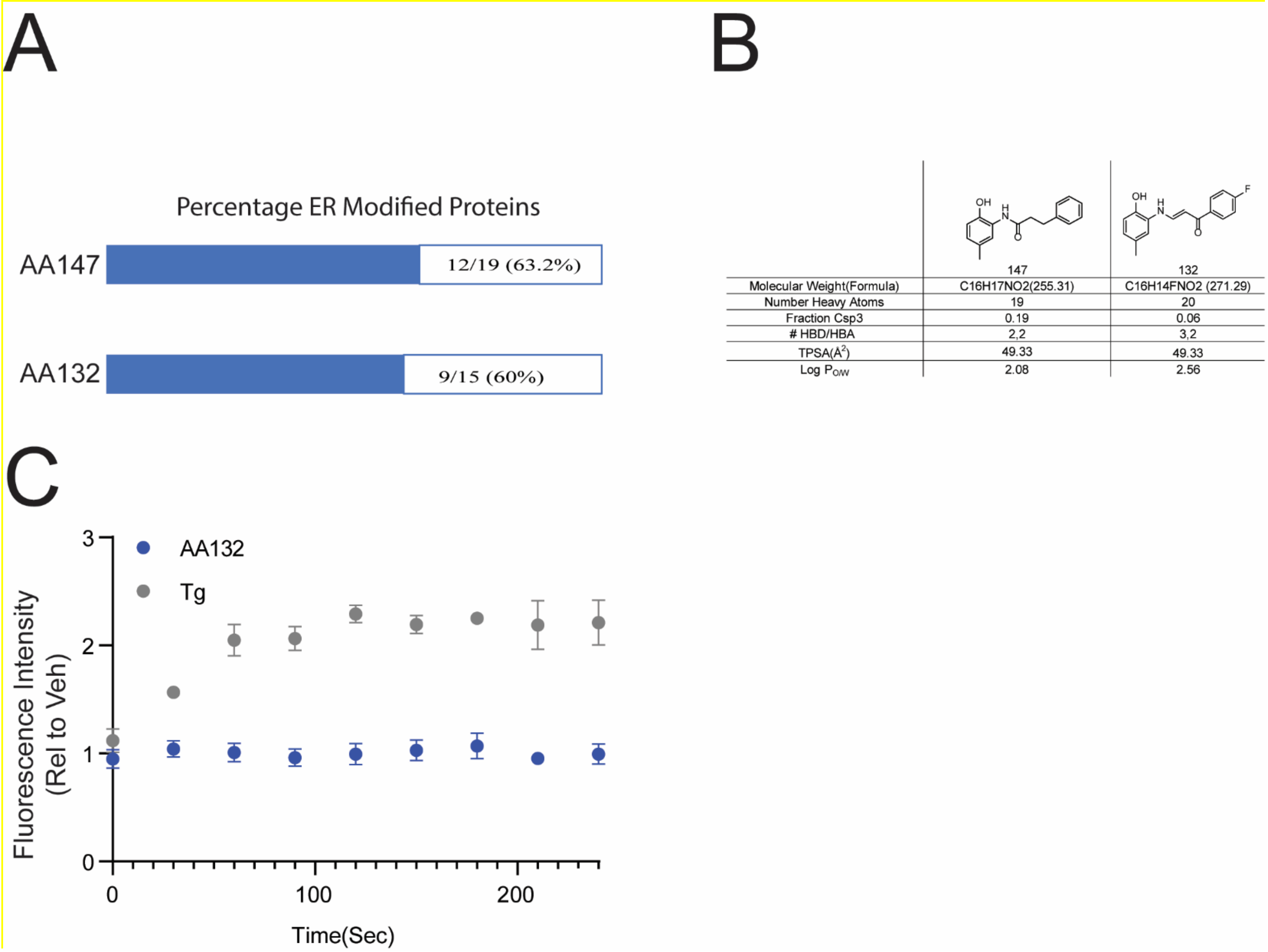

	147	132
Molecular Weight(Formula)	C16H17NO2(255.31)	C16H14FNO2 (271.29)
Number Heavy Atoms	19	20
Fraction Csp3	0.19	0.06
# HBD/HBA	2,2	3,2
TPSA($Å^2$)	49.33	49.33
Log $P_{O/W}$	2.08	2.56

Figure S3. AA132^{yne} Covalently Modifies ER PDIs. A. Graph showing proportion of AA147^{yne} and AA132^{yne} target proteins localized to the ER. **B.** Calculated physicochemical properties of AA147 and AA132**.** Values calculated using SwissADME (SwissADME.ch). TPSA = Total Polar Surface Area; HBD = Hydrogen Bond Donors; HBA = Hydrogen Bond Acceptors. **C.** Graph of change in fluorescence intensity of HEK293T cells stimulated with AA132 (10 µM) or Tg (500 nM) for indicated time points. Calcium mobilization detected using the Calcium No WashPLUS Detection Kit (DiscoveRx) according to the recommended protocol.

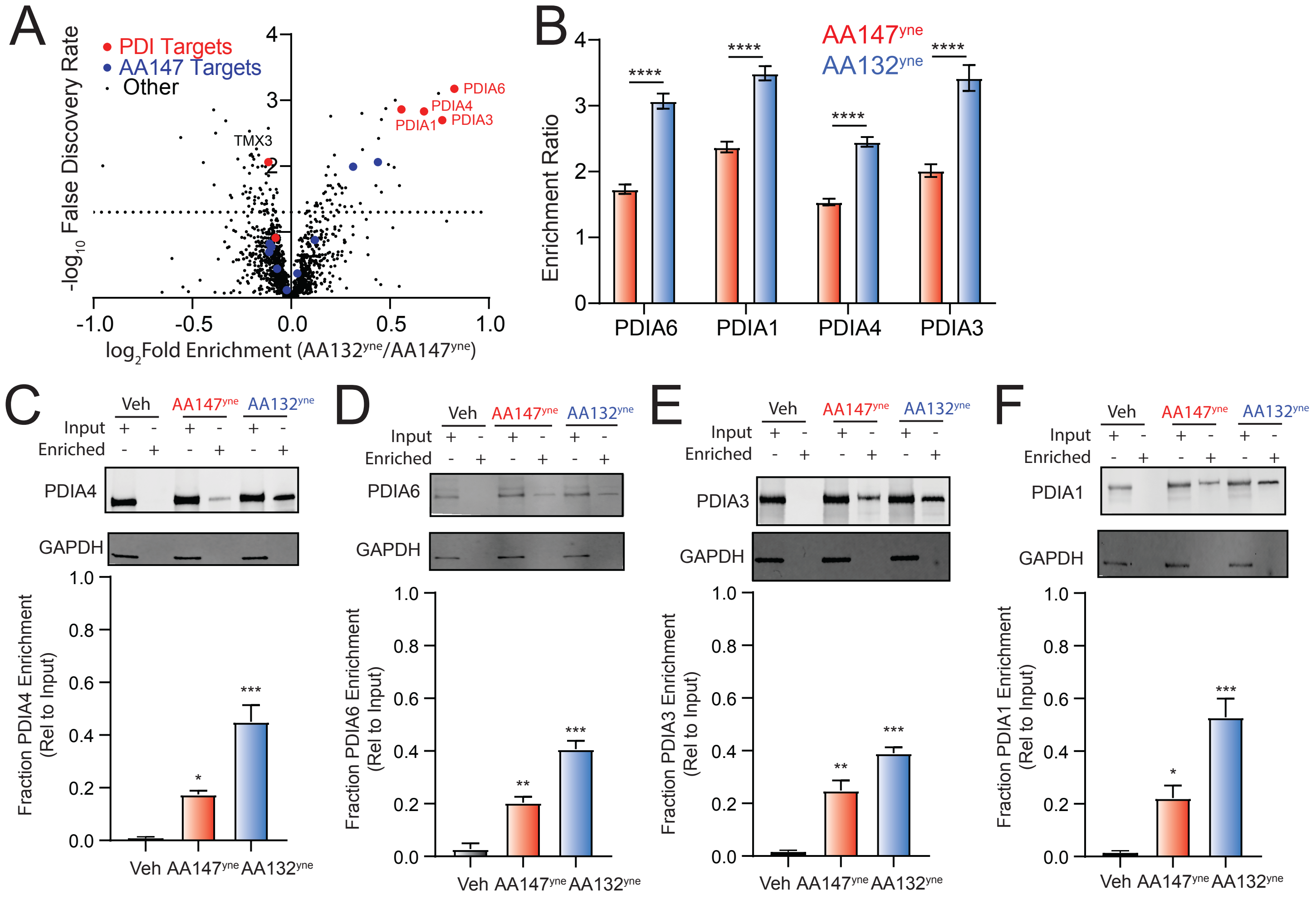

Figure S4. AA132^{yne} Shows Higher PDI Labeling as Compared to AA147^{yne}. A. Volcano plot showing $\log_2$ fold enrichment of AA132^{yne} labeled proteins relative to AA147^{yne} labeled proteins (x-axis) versus the –log FDR (y-axis) in HepG2 cells (10 µM, 6h). Proteins with GO annotation for PDI (GO: 0003756) labeled in red and additional previously defined AA147^{yne} targets labeled in blue. Data shown in **Table S2**. **B.** Bar graph of enrichment ratio of select PDIs by indicated the compound relative to DMSO from data shown in **Fig. S4A** (N = 4 biological replicates). ****p < 0.001 two-way ANOVA. **C-F**. Representative immunoblot and quantification of streptavidin-enriched PDIA4 (**C**), PDIA6 (**D**), PDIA3 (**E**), and PDIA1 (**F**) obtained from HEK293T cells treated with AA147^{yne} (10 µM; 6 h) or AA132^{yne} (10 µM; 6 h) and protein targets conjugated to biotin. Fraction enrichment was calculated by dividing the signal in enriched samples by the input signal. Error bars show SEM for n=3 replicates. *p < .05, **p<.01, ***p<.001 from one-way ANOVA relative to vehicle-treated cells.

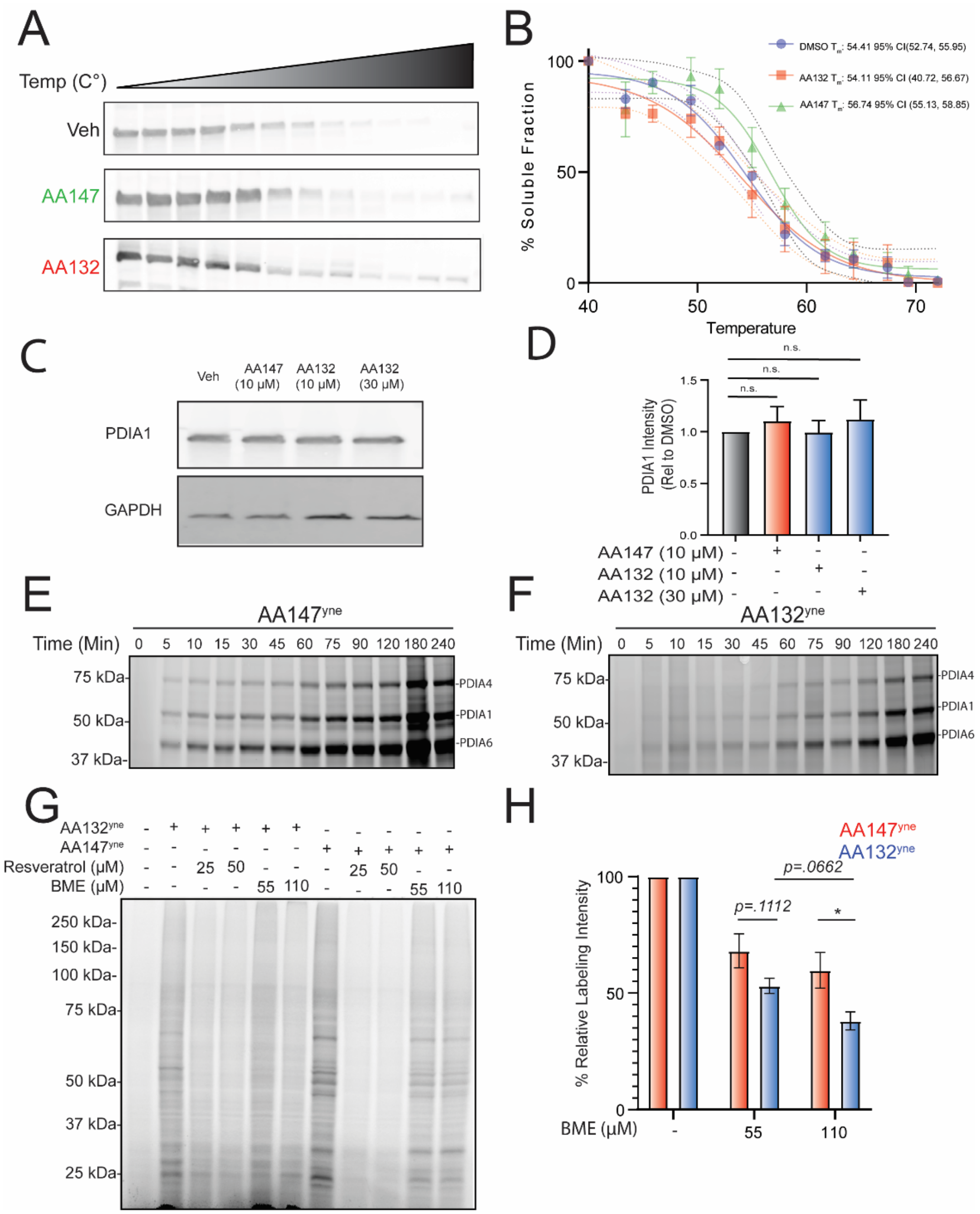

Figure S5. AA132^{yne} Shows Slower Protein Labeling Kinetics as Compared to AA147^{yne}. A. Representative immunoblot of the soluble fraction of PDIA1 from heat-treated ALMC2 cells at the temperatures indicated (40-72°C) preincubated with listed compound (10 µM, 2h). **B.** Graph of percent soluble fraction (y-axis) versus temperature (x-axis) for data in **Fig S5A**. Fitted curves calculated using Boltzmann Sigmoidal Fit in Prism and plotted with 95% confidence intervals (dotted lines). T_m is temperature on calculated sigmoidal curve with 50% soluble PDIA1 fraction remaining. Error bars represent S.E.M (N = 3 biological replicates). **C**. Representative immunoblot of PDIA1 levels from HEK293T cells treated with AA147(10 µM), AA132 (10 µM), or AA132 (30 µM) for 6h. **D.** Quantification of **Fig S5C**. Error bars represent standard error of the mean. **E.** Representative SDS-PAGE gel of Cy5-conjugated proteins from ALMC2 cells treated at indicated time point with AA147^{yne} (10 µM). **F.** Representative SDS-PAGE gel of Cy5-conjugated proteins from ALMC2 cells treated at indicated time point with AA132^{yne} (10 µM). **G.** Representative gel of AA132^{yne} and AA147^{yne} labeled proteins in HEK293T cells cotreated with indicated concentrations of β-mercaptoethanol or resveratrol for 4h. **H.** Quantification of **Fig S5G**. Error bars represent SEM for n=3 replicates. Percent labeling calculated as lane intensity relative to cotreatment with vehicle (0.1% DPBS) *$p < 0.05$ for a two-way ANOVA.

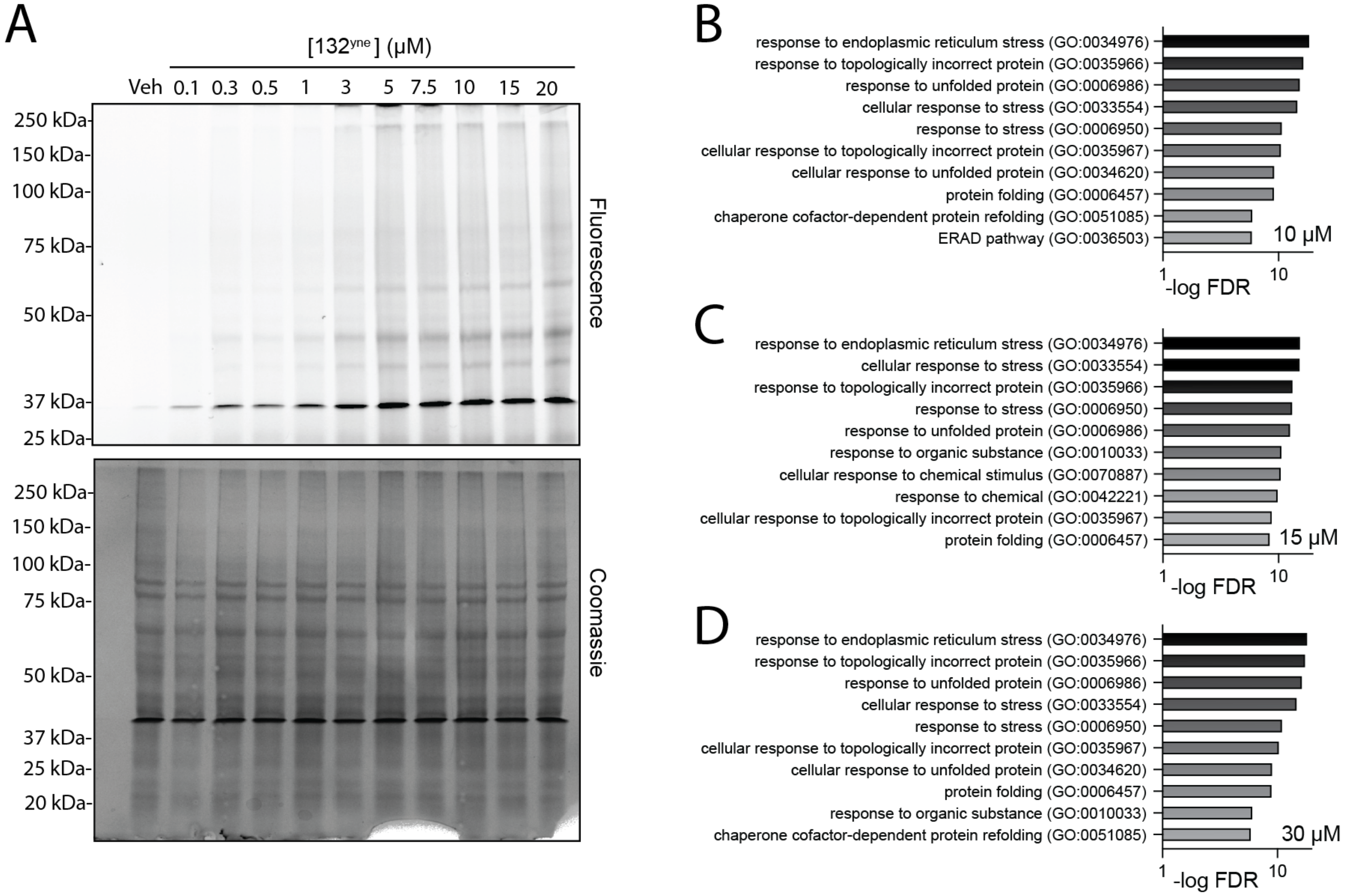

Figure S6. AA132 Selectively Activates ATF6 Transcriptional Signaling at Lower Doses. A. Fluorescence and coomassie-stained SDS-PAGE of lysates prepared from HEK293T cells treated with the indicated concentration of AA132^{yne} (4 h) and then conjugated to Cy-5.5-Azide. **B-D**. Top-10 GO terms for significantly induced genes (fold change >1.3, $p<0.05$) identified by RNAseq in HEK293T cells treated with 10 μM (**A**), 15 μM (**B**), or 30 μM (**C**) AA132 for 6 h. RNAseq data is included in **Table S3** Full GO analysis is included in **Table S4**.

SUPPLEMENTARY TABLE LEGENDS

Table S1. Excel spreadsheet showing the enrichment and competition ratio for proteins identified as targets of AA132^{yne}. Related to **Fig. 3.**

Table S2. Excel spreadsheet showing fold enrichment for AA132^{yne}/AA147^{yne} of proteins identified in proteomics experiments performed in HEK293T or HepG2 cells. Related to **Fig. 4** and **Fig. S4**.

Table S3. Excel spreadsheet showing DESeq outputs for HEK293T cells treated with increasing doses of AA132 or AA147. Related to **Fig. 6**.

Table S4. Excel spreadsheet showing GO analysis for RNAseq data for HEK293T cells treated with the indicated concentration of AA132. Related to **Fig. 6**.

Table S5. Excel spreadsheet showing the expression of transcriptional targets of ATF6, IRE1/XBP1s and PERK signaling from RNAseq data of HEK293T cells treated with the indicated concentration of AA132 or AA147. Related to **Fig. 6**.

SUPPLEMENTAL MATERIALS AND METHODS

Cellular Thermal Shift Assay

ALMC2 cells grown to concentration of 2 million cells/mL and 15 mL cell suspension incubated in T75 flask with indicated compound (10 µM, 2h, 37° C). After treatment, cells were pelleted by centrifugation (3 min, 300 g) and washed with PBS before resuspension in PBS at 30 million cells/mL. 100 µL cell suspension was added to 0.2 mL PCR tube before heat treatment at indicated temperature for 3 min and room temperature incubation for 3 min. Samples snap frozen at -80 C were lysed by sequential freeze-thaw cycles and centrifuged (15,000 g, 10 min) to pellet insoluble material. Soluble fractions were boiled for 5 min in Laemmli buffer with 100 mM DTT before loading onto SDS-PAGE gels. Proteins were transferred from gel slabs to PVDF membranes and blotted using rabbit anti-PDIA1 antibody (1:1000) Protein Tech) and visualized on the Odyssey Infrared Imaging System (Li-Cor Biosciences).

Chemoproteomic Analysis

HEK293T cells in 10 cm plates at 80-90% confluency were treated for 6 h with vehicle (0.1% DMSO), AA132^{yne} (10 µM), or the combination of AA132^{yne} (10 µM) and AA132 (40 µM) at 37 °C. The cells were washed with PBS before harvesting with tryspin, pelleting (500 g, 5 min), and washed with PBS (1 mL). Cells pellets were resuspended in radioimmunoprecipitation assay (RIPA) buffer before sonication with a probe tip sonicator to lyse the cells (15 sec, 3 sec on/2 off, 30% amplitude). For each sample, 1 g lysate (500 µL) were reacted with click reagents to give final concentrations as follows: 100 µM of diazo biotin-azide (Click Chemistry Tools, Scottsdale, AZ), 800 µM copper (II) sulfate, 1.6 mM BTTAA ligand (2-(4-((bis((1-tert-butyl-1H-1,2,3-triazol-4-yl)methyl)amino)methyl)-1H-1,2,3-triazol-1-yl)acetic acid) (Albert Einstein College), and 5 mM sodium ascorbate. The reaction was placed on a shaker at 1000 rpm at 30 °C for 90 The reaction was quenched with the sequential addition of cold methanol (4x volume), chloroform (1x volume), and DPBS (4x volume) to precipitate proteins. Proteins were pelleted by centrifugation (4,700 g, 10 min, 4 ˚C). The supernatant was discarded, and the pellets dried under

air for 5 min. Protein pellets were resuspended in 6M urea in PBS (500 μL) with brief sonication. 50 μL of high-capacity streptavidin beads were washed with PBS and mixed with the protein solution in 6 mL of phosphate-buffered saline (PBS). This suspension was placed on a rotator or a shaker and agitated for 2 h. The beads were centrifuged and washed 5 times with PBS and 1% SDS. The protein was eluted from the beads by two washes of 50 mM sodium dithionite in 1% SDS for 1 h and then precipitated by chloroform/methanol precipitation as described above. 50 μL of freshly made 1:1 mixture 200 mM TCEP·HCl in DPBS and 600 mM K_2CO3 in DPBS was added to each sample before incubation at 37 °C for 30 minutes while shaking. Alkylation of reduced thiols was achieved by addition of 70 μL freshly prepared 400 mM iodoacetamide in DPBS and incubation at room temperature while protected from light. The reaction was quenched by adding 130 μL of 10% SDS in DPBS and then diluted to approximately 0.2% SDS via DPBS (5.5 mL) and incubated with preequilibrated streptavidin agarose beads (3x1 mL PBS wash). The samples were rotated at room temperature for 1.5 hours, centrifuged at 2000 rpm for 2 minutes, and then washed sequentially with 5 mL 0.2% SDS in DPBS, 5 mL DPBS, and 5 mL 100 mM TEAB (Thermo Cat #90114) pH 8.5 to remove non-binding proteins. The beads were transferred to low-bind 1.5 mL Eppendorf tubes and the bound proteins digested overnight at 37 °C in 200 μL 100 mM TEAB containing 2 μg sequencing grade porcine trypsin, 1 mM CaCl2, and 0.01% ProteaseMax (Promega Cat #V2071). The beads were centrifuged at 2000 rpm for 5 minutes to separate the beads from the supernatant. 200 μL supernatant was transferred to a new tube using a gel-loading tip, and the beads were washed with 100 μL TEAB buffer. The beads were centrifuged at 2000 rpm for 5 minutes and the supernatant combined with the previous. 120 μL acetonitrile was added to each supernatant sample before addition of 80 μL (200 μg) of TMT 10 plex (Thermo Scientific, cat #90110) reconstituted in acetonitrile. The samples were incubated at room temperature for 1 hour and vortexed occasionally. 7 μL of freshly prepared 5% hydroxylamine in water was added to each sample to quench the reaction, vortexed, and incubated for 15 minutes before quenching with addition of 5 μL MS-grade formic acid. The samples were then vacuum centrifuged to dryness. The samples were

combined by redissolving the contents of one tube in 200 µL 0.1 % trifluoroacetic acid solution in water and sequential transfer to the respective multiplexed experiment until all samples were redissolved. This stepwise process was repeated with an additional 100 µL 0.1 % TFA solution for a final volume of 300 µL. The pooled samples were fractionated using the Pierce high pH Reversed-Phase Fractionation Kit (Thermo Fisher Scientific 84868) according to manufacturer's instructions. The peptide fractions were eluted from the spin column with solutions of 0.1% triethylamine containing an increasing concentration of MeCN (5 - 95% MeCN; 8 fractions). Samples were dried via vacuum centrifugation, reconstituted in 50 µL 0.1% formic acid, and stored at -80 °C until ready for mass spectrometry analysis.

Affinity precipitation and quantitative TMT-MuDPIT analysis of proteins covalently modified by AA147^{yne} and AA132^{yne}

HEK293T-Rex or HepG2 cells in 10 cm plates were treated for 6 h with vehicle (0.1% DMSO), AA147^{yne} (10 µM), or AA132^{yne} (10 µM), at 37 °C. Lysates were prepared in radioimmunoprecipitation assay (RIPA) buffer (150 mM NaCl, 50 mM Tris pH 7.5, 1% Triton X-100, 0.5% sodium deoxycholate, and 0.1% SDS) with fresh protease inhibitor cocktail (Roche, Indianapolis, IN) and centrifuged for 20 min at 10000×g. Protein concentrations of supernatants were determined by the BCA assay (Thermo Fisher). For each sample, 100 µg of lysate were reacted with click reagents to give final concentrations as follows: 100 µM of diazo biotin-azide (Click Chemistry Tools, Scottsdale, AZ), 800 µM copper (II) sulfate, 1.6 mM BTTAA ligand (2-(4-((bis((1-tert-butyl-1H-1,2,3-triazol-4-yl)methyl)amino)methyl)-1H-1,2,3-triazol-1-yl)acetic acid) (Albert Einstein College), and 5 mM sodium ascorbate. The reaction was placed on a shaker at 1000 rpm at 30 °C for 2 h. The proteins were then precipitated from the reaction mixture by adding an equal volume of 3:1 chloroform/methanol. The pellet was washed three times with 1:1 chloroform/methanol. The precipitate was suspended in 500 µL of 6 M urea with 25 mM ammonium bicarbonate and 140 µL of 10% SDS was added to this mixture to help solubilize the protein. 50 µL of

high-capacity streptavidin beads were washed with PBS and mixed with the protein solution in 6 mL of phosphate-buffered saline (PBS). This suspension was placed on a rotator or a shaker and agitated for 2 h. The beads were centrifuged and washed 5 times with PBS and 1% SDS. The protein was eluted from the beads by two washes of 50 mM sodium dithionite in 1% SDS for 1 h and then precipitated by chloroform/methanol precipitation as described above.

Air-dried pellets from the affinity precipitation were resuspended in 1% RapiGest SF (Waters) in 100 mM HEPES (pH 8.0). Proteins were reduced with 5 mM tris(2-carboxyethyl)phosphine hydrochloride (Thermo Fisher) for 30 min and alkylated with 10 mM iodoacetamide (Sigma Aldrich, St. Louis, MO) for 30 min at ambient temperature and protected from light. Proteins were digested for 18 h at 37 °C with 2 µg trypsin (Promega). After digestion, 20 µg of peptides from each sample were reacted for 1 h with the appropriate TMT-NHS isobaric reagent (ThermoFisher) in 40% (v/v) anhydrous acetonitrile and quenched with 0.4% NH_4HCO_3 for 1 h. Samples with different TMT labels were pooled and acidified with 5% formic acid. Acetonitrile was evaporated on a SpeedVac and debris was removed by centrifugation for 30 min at 18,000×g. MuDPIT (Multi-Dimensional Protein Identification Technology) microcolumns were prepared as described previously 79. LCMS/MS analysis was performed using a Q Exactive mass spectrometer equipped with an EASY nLC 1000 (Thermo Fisher). MuDPIT experiments were performed by 5 min sequential injections of 0, 20, 50, 80, 100% buffer C (500 mM ammonium acetate in buffer A) and a final step of 90% buffer C / 10% buffer B (20% water, 80% acetonitrile, 0.1% fomic acid, v/v/v) and each step followed by a gradient from buffer A (95% water, 5% acetonitrile, 0.1% formic acid) to buffer B. Electrospray ionization was performed directly from the analytical column by applying a voltage of 2.5 kV with an inlet capillary temperature of 275°C. Data-dependent acquisition of MS/MS spectra was performed with the following settings: eluted peptides were scanned from 400 to 1800 m/z with a resolution of 30,000 and the mass spectrometer in a data dependent acquisition mode. The top ten peaks for each full scan were fragmented by HCD using a normalized collision energy of 30%, a 100 ms activation time, a resolution of 7500, and scanned from

100 to 1800 m/z. Dynamic exclusion parameters were 1 repeat count, 30 ms repeat duration, 500 exclusion list size, 120 s exclusion duration, and exclusion width between 0.51 and 1.51. Peptide identification and protein quantification was performed using the Integrated Proteomics Pipeline Suite (IP2, Integrated Proteomics Applications, Inc., San Diego, CA) as described previously.

General Synthetic Procedures

All compounds and reagents were purchased from Sigma-Aldrich, Acros, Alfa Aesar, Combi-blocks, and EMD Millipore unless otherwise noted and were used without further purification. Thin layer chromatography with Merck silica plates (60-F254), using UV light as the visualizing agent, was used to monitor reaction progress. Flash column chromatography was carried out using a Teledyne Isco Combiflash Nextgen 300+ machine using Luknova SuperSep columns (SiO_2,25 μm) with ethyl acetate and hexanes as eluents. ^{1}H NMR spectra were recorded on a Varian INOVA-400 400MHz spectrometer. Chemical shifts are reported in δ units (ppm) relative to residual solvent peak. Coupling constants (*J*) are reported in hertz (Hz). Characterization data are reported as follows: chemical shift, multiplicity (s=singlet, d=doublet, t=triplet, q=quartet, br=broad, m=multiplet), coupling constants, number of protons, mass to charge ratio. The compound's identity was confirmed via high-resolution mass spectrometry.

Synthesis of AA132

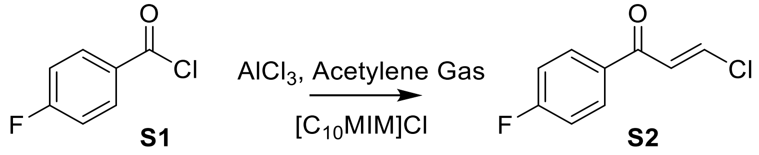

To a flame dried flask added 1-Decyl-3-methylimidazolium chloride (3.52 g, 13.6 mmol, 3.2 eq) and aluminum chloride (1.02 g, 7.65 mmol, 1.8 eq) and stirred for 16 hours under Argon to make the chloroaluminate fluid. To this flask at 0 °C, added 4-fluorobenzoyl chloride (S1, 500 μL, 4.25 mmol, 1

eq) slowly and the mixture became syrupy. The mixture was warmed to 75 ° C and acetylene gas generated in a separate flask from calcium carbide was bubbled through for 2 hours to afford the ß-chlorovinyl ketone. The crude was added to ice water and extracted with ether which was washed once with brine and concentrated to give a yellow oil (S2), which was used for the next step without further purification.

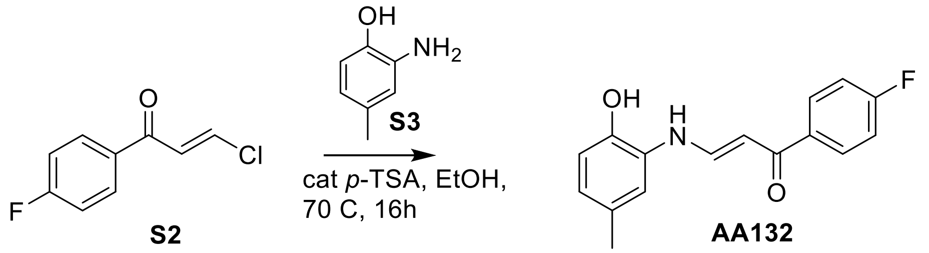

To a microwave vial with S2 (141 mg, 0.75 mmol, 1 eq), we added 2-amino-*p*-cresol (S3, 91 mg, 0.75 mmol, 1 eq) and *p*-toluenesulfonic acid (28.5 mg, 0.15 mmol,0.2 eq). Solids were dissolved in 3 mL EtOH and stirred under argon for 16h at 70 °C. Reaction diluted in EtOAc and washed sequentially with water and 1M HCl before drying over $MgSO_4$. Product purified by column chromatography (SiO_2, 4:1 Hex:EtOAc) to give AA132 as a yellow solid (98.2 mg, 48% yield).

^{1}H NMR (400 MHz, Acetone) δ 12.28 (d, J = 12.8 Hz, 1H), 8.82 (d, J = 0.6 Hz, 1H), 8.13 – 8.01 (m, 2H), 7.86 (ddd, J = 12.8, 7.8, 0.5 Hz, 1H), 7.29 – 7.19 (m, 3H), 6.88 (d, J = 8.1 Hz, 1H), 6.74 (dtd, J = 8.0, 1.4, 0.7 Hz, 1H), 6.13 (d, J = 7.8 Hz, 1H), 2.28 (d, J = 0.7 Hz, 3H).

^{19}F NMR (376 MHz, Acetone) δ -113.86

^{13}C NMR (100 MHz, ACETONE-D6) δ 188.04, 165.98, 163.50, 144.29, 143.67, 136.15, 136.12, 129.89, 129.80, 129.78, 128.54, 123.92, 115.44, 115.28, 115.07, 114.47, 92.78, 20.07.

HRMS: calculated for $C_{16}H_{14}FNO_2$[M+ H^+] 272.1087, found 272.1085

Synthesis of AA132^{yne}

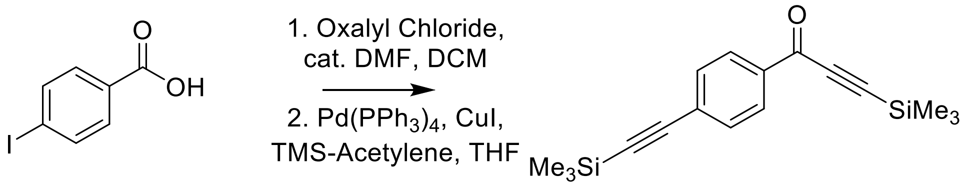

S3 **S4**

To 4-Iodobenzoic acid (S3, 738 mg, 3 mmol, 1 eq) dissolved in 15 mL DCM added oxalyl chloride (321 µL, 3.75 mmol, 1.25 eq) dropwise at 0 °C before addition of several drops of DMF. Reaction allowed to warm to room temperature and stir for 3h before TLC indicated complete conversion to the acyl chloride. Solvent removed under reduced pressure to give a yellow-white powder. To flask containing crude residue added copper iodide (50 mg, .11 mol%) and Palladium-tetrakis(triphenylphosphine) (80 mg, 2 mol %), then dissolved in 10 mL anhydrous THF. After 3x freeze-pump-thaw cycles, added diisopropylethylamine (2.6 mL, 15 mmol, 5 eq), and trimethylsilylacetylene (980 µL, 3.3 mmol, 1.1 eq). Reaction stirred under argon at 40 °C overnight before washing with saturated $NaHCO_3$, and brine before drying over $MgSO_4$ and concentrating. The crude residue was purified by flash column chromatography (SiO_2, 9:1 Hex/EtOAc) to give an orange solid (S4**,** 638 mg, 71 % yield).

^{1}H NMR (400 MHz, Acetone) δ 8.18 – 8.08 (m, 2H), 7.70 – 7.61 (m, 2H), 0.35 (d, J = 1.1 Hz, 9H), 0.28 (d, J = 1.0 Hz, 9H).

^{13}C NMR (100 MHz, Acetone-D6) δ 175.96, 136.06, 132.20, 129.41, 128.95, 103.96, 100.51, 98.61, -1.02, -1.49.

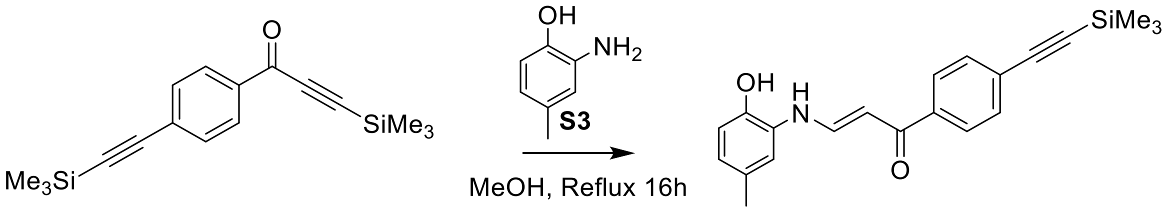

S4 **S5**

To a microwave vial charged with S4 (59 mg, 0.2 mmol, 1 eq), we added 2-amino-*p*-cresol (S3**;** 23.6 mg, 0.2 mmol, 1 eq). The reaction vessel was sealed before addition of 1 mL anhydrous methanol and heated at 70 ° C for 16 h. Reaction diluted in EtOAc, washed with 1M HCl and brine, and dried over $MgSO_4$. The solvent was removed under reduced pressure. The crude residue purified by flash column chromatography (SiO_2, 9:1 Hex/EtOAc) to afford the product S5 as a yellow powder (43 mg, 62% yield).

^{1}H NMR (500 MHz, Acetone) δ 12.36 (d, *J* = 12.8 Hz, 1H), 8.86 (s, 1H), 8.03 – 7.97 (m, 2H), 7.93 – 7.85 (m, 1H), 7.58 – 7.54 (m, 2H), 7.25 (d, *J* = 1.8 Hz, 1H), 6.88 (d, *J* = 8.1 Hz, 1H), 6.75 (ddd, *J* = 8.1, 2.0, 0.8 Hz, 1H), 6.16 (d, *J* = 7.8 Hz, 1H), 2.28 (d, *J* = 0.7 Hz, 3H), 0.27 (s, 9H).

^{13}C NMR (126 MHz, Acetone) δ 205.25, 188.20, 144.44, 143.67, 139.40, 131.72, 129.73, 128.42, 127.27, 127.25, 125.74, 123.99, 115.42, 114.52, 104.58, 96.07, 93.00, 28.79, 28.64, 28.48, 19.99, -1.02.

HRMS: calculated for $C_{17}H_{22}OSi_2$ [M+ H$^+$] 299.1287, found 299.1289

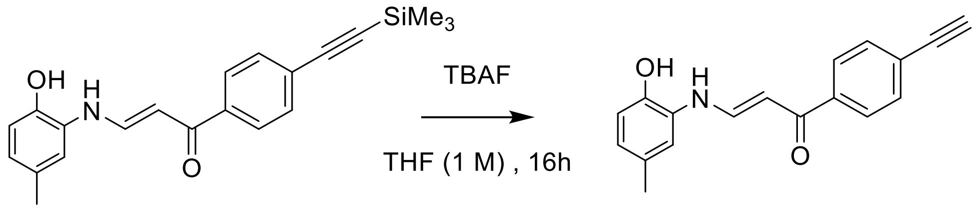

S5 **AA132^{yne}**

To S5 (0.1 mmol, 35 mg) dissolved in 0.5 mL THF, we added 0.5 mL of tetrabutylammonium fluoride (1 M) in THF overnight dropwise. Reaction was allowed to stir overnight at room temperature. Reaction

diluted in EtOAc and washed with 1M HCl. The organic layer was collected, dried over $MgSO_4$, and solvent removed under reduced pressure. The crude was purified by flash column chromatography (SiO_2 4:1 Hex: EtOAc) to afford the product AA132^{yne} as a yellow powder (23.3 mg, 86 % yield).

^{1}H NMR (400 MHz, ACETONE-D_6) δ 12.32 (d, J = 12.9 Hz, 1H), 8.89 (s, 1H), 8.01 – 7.90 (m, 2H), 7.85 (dd, J = 12.8, 7.8 Hz, 1H), 7.59 – 7.53 (m, 2H), 7.21 (d, J = 1.9 Hz, 1H), 6.84 (d, J = 8.1 Hz, 1H), 6.76 – 6.67 (m, 1H), 6.12 (d, J = 7.7 Hz, 1H), 3.81 (s, 1H), 2.23 (s, 3H).

^{13}C NMR (100 MHz, ACETONE-D_6) δ 205.44, 188.31, 144.55, 143.75, 139.62, 132.00, 129.77, 128.44, 127.37, 125.09, 124.09, 115.47, 114.56, 93.05, 82.99, 80.54, 20.08.

HRMS: calculated for $C_{18}H_{15}NO_2$ [M+ H$^+$] 278.1181, found 278.1187

NMR Spectra for AA132

^{1}H NMR for AA132

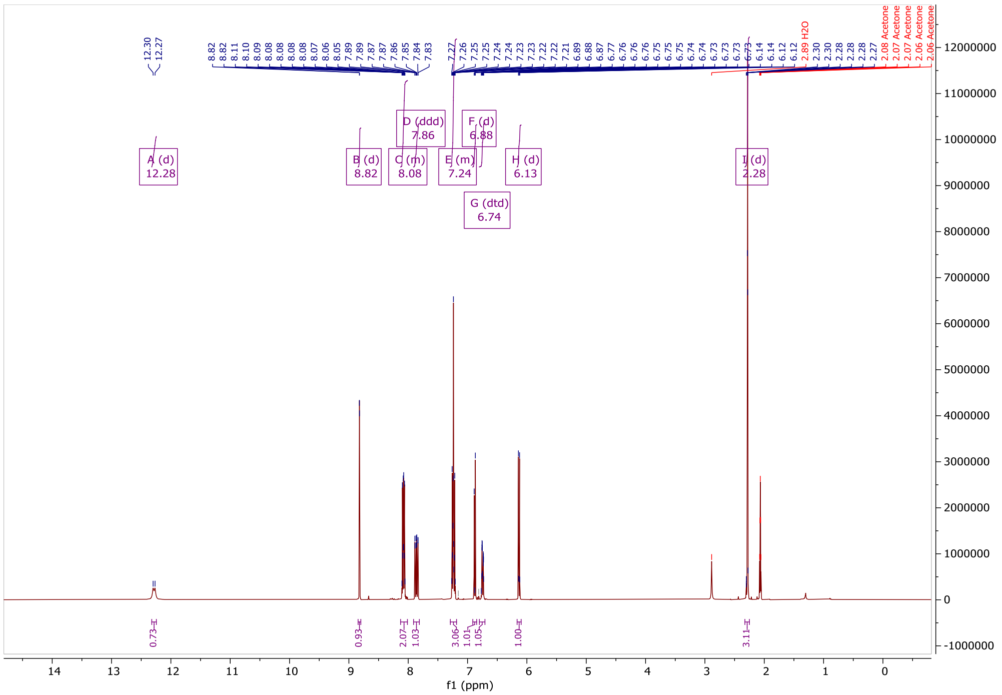

^{13}C NMR for AA132

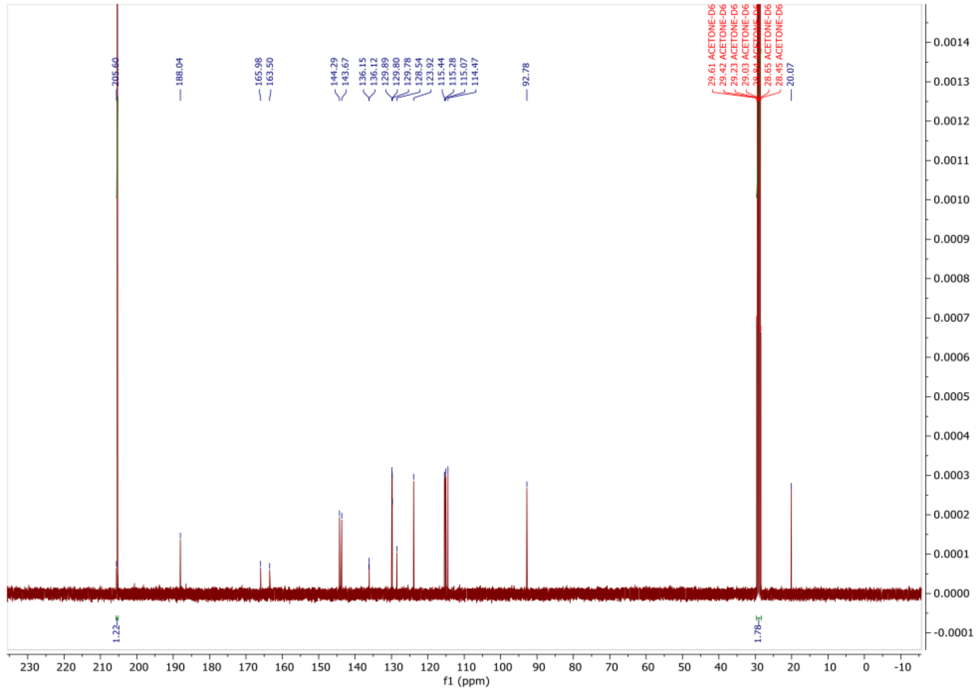

^{19}F NMR spectra for AA132

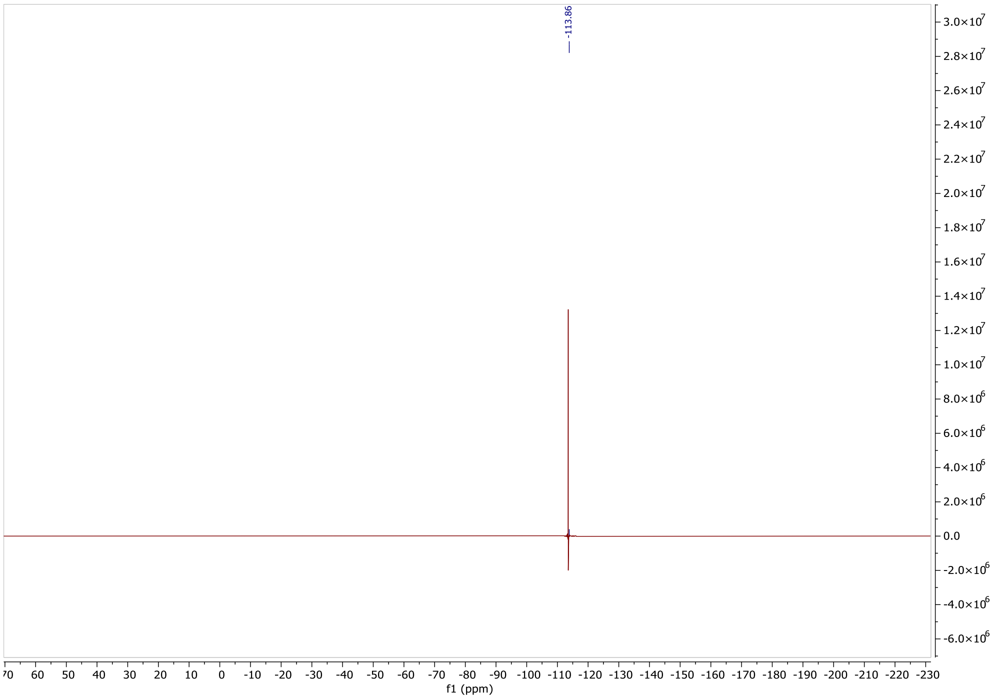

NMR Spectra for S5

^{1}H NMR for S5

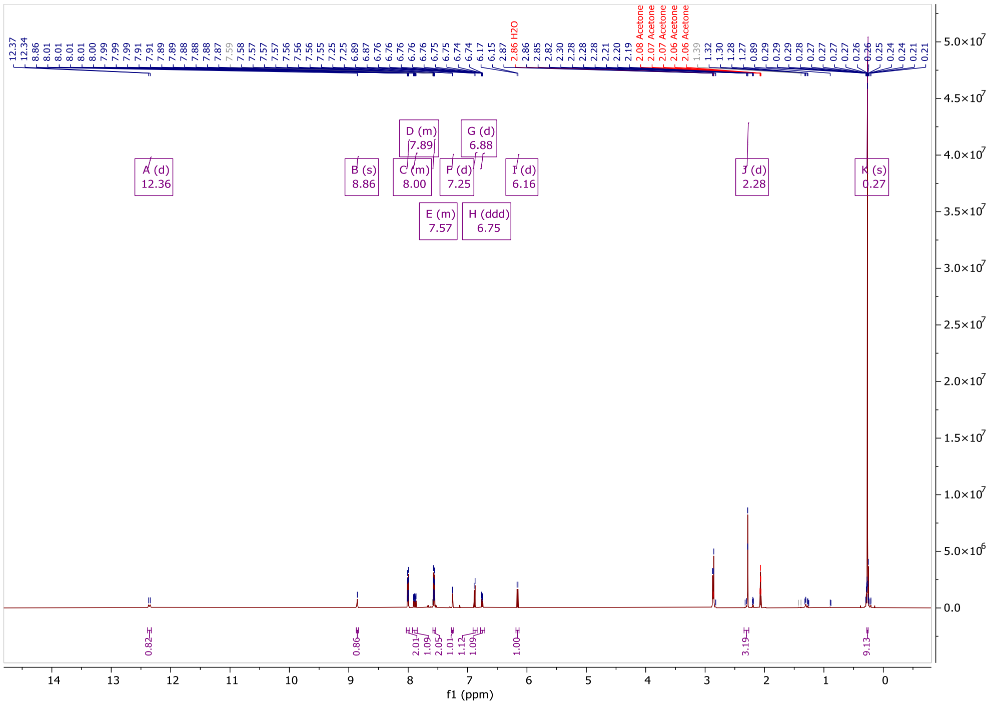

^{13}C NMR for S5

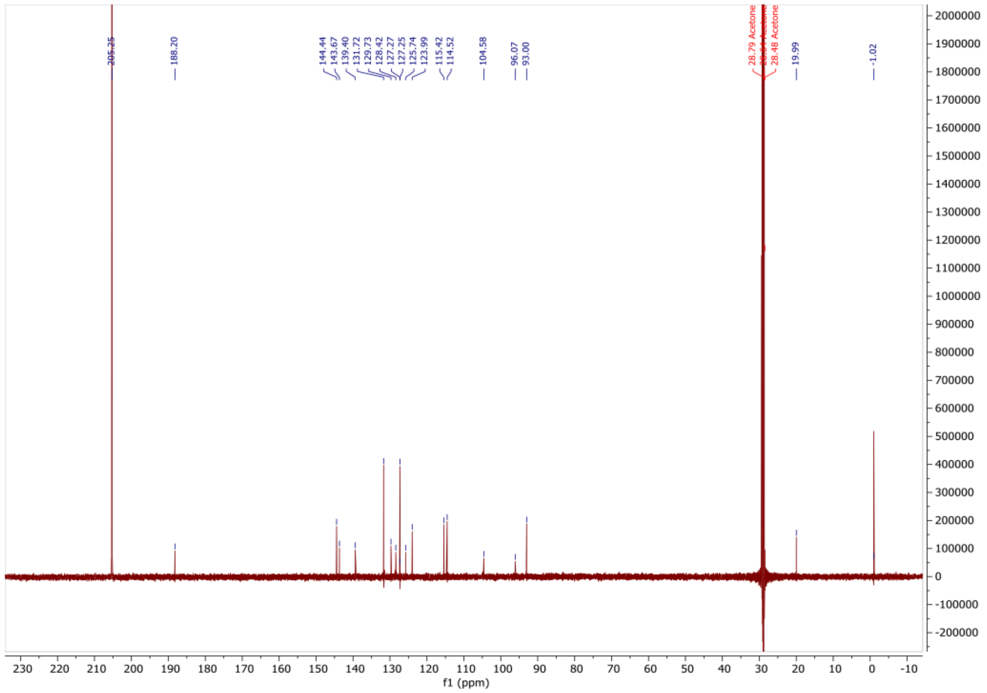

NMR Spectra for AA132^{yne}

^{1}H NMR for AA132^{yne}

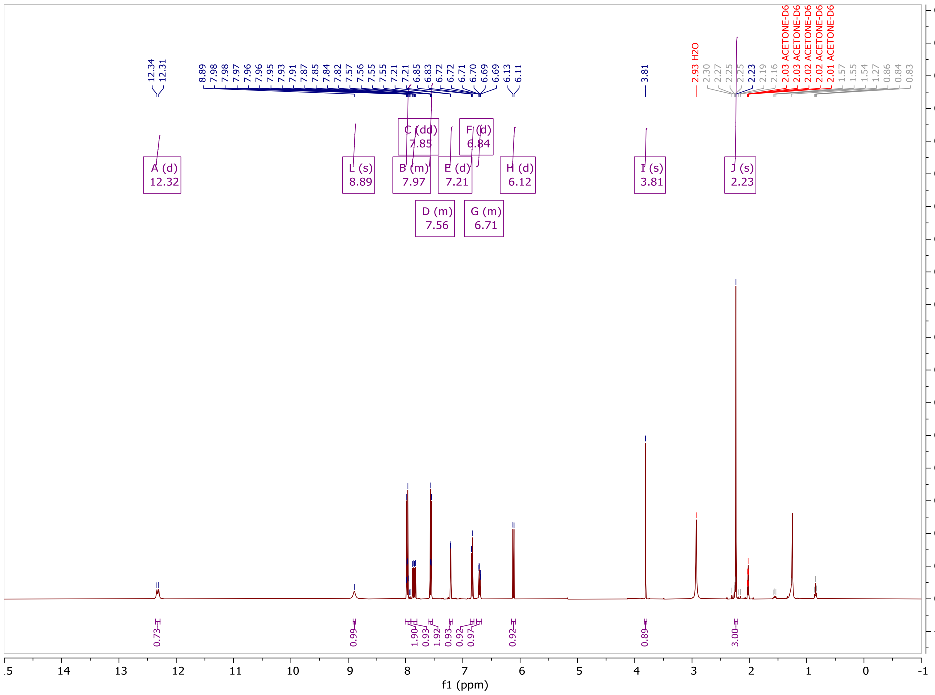
A (d)
12.32
L (s)
8.89
B (m)
7.97
C (dd)
7.85
D (m)
7.56
E (d)
7.21
F (d)
6.84
G (m)
6.71
H (d)
6.12
I (s)
3.81
J (s)
2.23
2.93 H2O
2.03 ACETONE-D6
2.03 ACETONE-D6
2.02 ACETONE-D6
2.02 ACETONE-D6
2.01 ACETONE-D6
f1 (ppm)

^{13}C NMR for AA132^{yne}

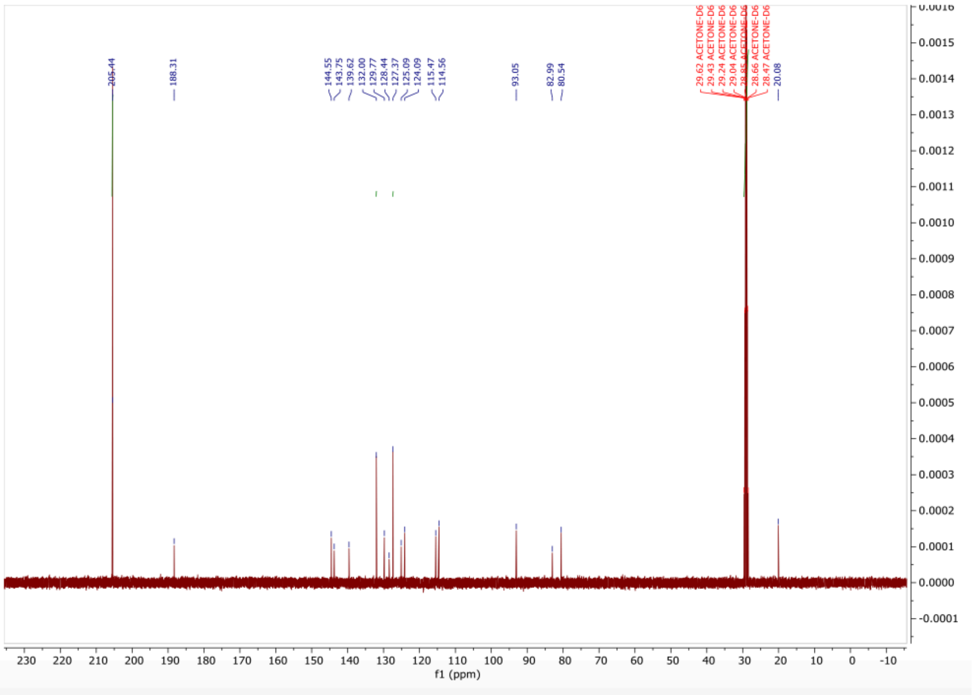

Supplement: Supplementary file 1 — cb3c00042_si_001.pdf [file cb3c00042_si_001.pdf]
